# Acupoint Catgut Embedding for Insomnia: A Meta-Analysis of Randomized Controlled Trials

**DOI:** 10.1155/2020/5450824

**Published:** 2020-11-06

**Authors:** Wanrong Li, Zhen Li, Huixing Zhang, Yue Wang, Hui Chen, Lize Xiong

**Affiliations:** Department of Anesthesiology and Perioperative Medicine, Translational Research Institute of Brain and Brain-Like Intelligence, Shanghai Fourth People's Hospital Affiliated to Tongji University School of Medicine, Shanghai 200434, China

## Abstract

**Objectives:**

A Meta-analysis was carried out to evaluate the efficacy and safety of acupoint catgut embedding (ACE), a procedure of embedding sutures made of absorbable materials into the skin tissue of acupoints, on insomnia.

**Methods:**

Relevant clinical randomized controlled trials (RCTs) were comprehensively searched from eleven electronic databases (up to 1 March 2020). Two authors independently screened literature, extracted data, and assessed the risk of bias of included studies. Stata 12 and RevMan 5.3.0 software were used for meta-analysis. PyCharm 2019 and Gephi software (version 0.9.2) were used for complex network analysis.

**Results:**

Thirty-four RCTs involving 2,655 patients were included. The meta-analysis suggested that ACE induced a better clinical efficacy compared with that in the estazolam tablets (EZ) group (RR = 1.22, 95% CI: 1.13, 1.31) or in the acupuncture (ACU) group (RR = 1.21, 95% CI: 1.14, 1.28) and could significantly reduce the score of Pittsburgh Sleep Quality Index (*P* < 0.05). ACE resulted in better long-term efficacy compared to that in the EZ group (RR = 1.87, 95% CI: 1.58, 2.22) and ACU group (RR = 1.30, 95% CI: 1.14, 1.48). ACE could significantly reduce the incidence of adverse events (RR = 0.30, 95% CI: 0.15, 0.60) compared with that in the EZ group. Complex network analysis indicated that acupoints of BL23, SP6, PC6, BL15, BL20, BL18, and HT7 were the core acupoints selected in ACE for insomnia.

**Conclusion:**

The clinical efficacy of ACE for insomnia is better than that of other interventions (EZ and ACU) in both short-term and long-term observations. Considering the efficacy and reduced visits to the clinic by ACE, the present study provides a practical and convenient complementary and alternative therapy for insomnia. This trial is registered with PROSPERO CRD 42020169866.

## 1. Introduction

Sleep disorders, especially insomnia, have become an emerging global pandemic. World Health Organization estimates that more than 1/3 of the population worldwide are sleep deprived [1]. Epidemiological studies show that approximately 10% to 23% of adolescents and 15% to 40% of adults worldwide experience different degrees of insomnia with an increased prevalence among the elderly, females, and people with medical or psychiatric disorders [2–9]. In China, 38.2% of adults suffer from insomnia, which is well above the international level that the World Health Organization reports for adults worldwide, and more than 300 million Chinese suffer from sleep disorders [10].

Studies have found that insomnia is an important risk factor for various diseases, including cardiovascular diseases, depression, diabetes, a weakened immune system, suicidal tendencies, and other mental disorders [11–15]. Furthermore, insomnia also increases the socioeconomic burden with more than $100 billion a year lost due to poor workplace performance and more traffic and fall accidents [16, 17].

Fortunately, insomnia is a treatable sleep disorder and a modifiable risk factor for a variety of illnesses [18]. At present, treatment of insomnia mainly includes drug therapy, nondrug therapy, or a combination of both. However, drug therapies have adverse effects such as headache, dizziness, withdrawal reaction, bitter mouth, forgetfulness, lethargy, falls, and hangover [19]. These side effects may increase the risk of developing cancers such as oral cancer, liver cancer, and breast cancer [20]. The first-line nondrug therapy is cognitive behavioral therapy for insomnia (CBT-I) [21], but it may not be a suitable treatment for everyone [22]. It is usually practiced by a licensed psychologist trained in behavioral sleep medicine or by a master-level clinician. One of the main obstacles to the expansion of CBT in the treatment of insomnia is the lack of trained clinicians and the availability of experts outside urban centers [18]. Acupuncture, defined as the use of an acupuncture needle by hand manipulation, has been proved to be an effective treatment for insomnia [23–26]. However, it has some disadvantages, such as it usually needs to be performed once a day or every other day [27].

Acupoint catgut embedding (ACE) refers to the procedure of embedding sutures made of absorbable materials into the skin tissue of acupoints which are closely related to different physiological processes or diseases [27]. It is a combination of traditional acupuncture and modern tissue therapy [28]. As a form of acupuncture, ACE can persistently stimulate acupoints for a week or longer until sutures are absorbed through softening, liquefaction, and absorption [28]. Its advantages are the long duration of acupoint stimulation, fewer times of treatment and less expense, short treatment cycle, and being acceptable for patients, etc. It reduces the frequency of patients seeking medical treatment and saves medical resources to a certain extent. It is more convenient and easier to be performed than traditional acupuncture. Therefore, it is used to treat insomnia in China, and its efficacy for insomnia has been proved in previous clinical trials [29]. At the same time, a meta-analysis [30] found that acupoint catgut embedding is a potential alternative therapy for insomnia compared to estazolam tablets by analyzing results from 7 randomized controlled trials (RCTs). However, only Chinese databases were searched in their study. Another study [31] analyzed the effective rate of 22 RCTs, but not the Pittsburgh Sleep Quality Index (PSQI) score, and did not take different treatment methods in the control group into account. Therefore, it is necessary to confirm the efficacy of ACE for insomnia with more rigorous methodologies. Besides, a few questions remain to be answered: Is ACE better than drugs such as benzodiazepines? Is ACE better than traditional acupuncture? Is ACE is safer than other types of treatment for insomnia? What are the core acupoints to perform ACE for insomnia? The present study aimed to answer these questions by conducting a comprehensive updated meta-analysis.

## 2. Methods

The present study was registered with PROSPERO and was conducted by conforming to the PRISMA guidelines [32].

### 2.1. Searching Strategy

Eight databases including the Cochrane Library, PubMed, Embase, the Web of Science, SinoMed, VIP database, Wan-Fang Database, and the China National Knowledge Infrastructure (CNKI) were searched up to 1st March 2020 without language restrictions. The following three databases for prospectively registered and ongoing trials were also searched: World Health Organization International Clinical Trials Registry Platform, Clinical Trials, and the Chinese Clinical Trials Registry. In addition, references of included studies were also searched to identify other potential clinical trials. Searching terms (MeSH terms combined with free terms) included intervention methods (acupoint catgut embedding), diseases (insomnia), and types of research (randomized controlled trial) (see the detailed searching strategy in Supplementary file 1: Table S1). Equivalent searching terms were employed in Chinese databases. A manual search was conducted for eligible studies listed in references of certain studies.

### 2.2. Inclusion and Exclusion Criteria

Studies meeting the following criteria were included in the meta-analysis. (a) RCTs were included without restriction about whether using blind methods. (b) Individuals were diagnosed with insomnia, regardless of the subtype of insomnia, gender, age, duration of the disease, ethnicity, and education status; The diagnosis of insomnia needs to be consistent with the international classification of diseases or other guidelines for the diagnosis and treatment of insomnia in China [33–36]. (c) The intervention group received ACE while the control group received placebos, medications, sham ACE, no treatment, or acupuncture/electro-acupuncture. (d) The main outcome measures included clinical therapeutic effect and PSQI score.

Exclusion criteria included (a) studies that compare the effect of different catgut lengths, catgut placing procedure, or acupoints for ACE; (b) studies in which catgut embedding acupoints were not clearly reported in the intervention measures; (c) studies with incomplete or questionable data; and (d) repeated publications or duplicate data.

### 2.3. Study Selection and Data Extraction

Two authors (WL and ZL) independently retrieved and reviewed all the eligible studies and extracted data. Disagreements between two authors were resolved by discussions or consulting a third author (HZ). The following information was extracted from the included studies and expressed in an excel table: research details (name of the first author, title, and year of publication), participant details (age, course of the disease, diagnostic and exclusion criteria), interventions (sample sizes, random sequence generation, allocation concealment, blinding methods, selective reporting and other biases, interventions in treatment and control groups, acupoints for ACE, duration of treatment, frequency of ACE and interventions of the control group, number of ACE, and follow-up visits), and primary and secondary outcomes (clinical therapeutic effect, PSQI scores, sleep quality (SQ) scores, fall asleep time (FAT) scores, sleep time (ST) scores, sleep efficiency (SE) scores, sleep disorder (SD) scores, daytime dysfunction (DD) scores, hypnotic drug (HD) scores, and adverse events).

We also considered the possible different types of missing data. First, we contacted the authors of the included studies to obtain the missing data. Second, we dealt with missing studies (and the associated risk of bias) by assessing for publication bias through the nonparametric trim-and-fill methods. The studies which did not report on either of our primary outcomes were excluded. The studies with missing data were also excluded for those we did not receive a corresponding response from study authors.

### 2.4. Quality Assessment

Two authors (HZ and YW) independently used the Cochrane Collaboration's risk of bias tool [37] to evaluate each included study, and differences between the two were resolved through discussion with a third author (WL). The following domains were assessed: randomization sequence generation, allocation concealment, blinding of participants and personnel, blinding of outcome assessment, incomplete outcome data, selective reporting, and other biases. According to the quality assessment tool, each study was rated as low risk, high risk, or unknown risk, respectively.

### 2.5. Statistical Analysis

Stata 12 and RevMan 5.3.0 software were used for meta-analysis. PyCharm 2019 and Gephi (version 0.9.2) were used for complex network analysis. The dichotomous data used RR and 95% confidence interval (CI) as the statistical indicator. The standardized mean difference (SMD) and 95% CI were used for continuous data.

The Forest plot was built by considering *P* < 0.05 as statistically significant. Statistical heterogeneity was measured using the chi-squared test and *I*^2^ statistics. For the chi-squared test, *P* < 0.1 indicated statistical significance. And the *I*^2^ values of 25%, 50%, and 75%, respectively, denoted cut-off points for low, moderate, and high levels of heterogeneity [38]. If a low or moderate statistical heterogeneity was found (*P* > 0.1, *I*^2^ < 50%), the fixed-effect model was employed. When significant heterogeneity was observed in the studies (*P* < 0.1, *I*^2^ ≥ 50%), a random-effect model was used to analyze the data. Sensitivity analysis, univariate metaregression analysis, and subgroup analysis were carried out to explore sources of potential heterogeneity and examine the robustness of primary results [39]. A publication bias analysis was performed when more than 10 studies were pooled. Publication bias was measured with a funnel plot and Egger's test [40]. The nonparametric trim-and-fill methods [41] were utilized to further evaluate the potential publication bias.

## 3. Results

### 3.1. Searching Results and Study Selection

**Figure 1:**
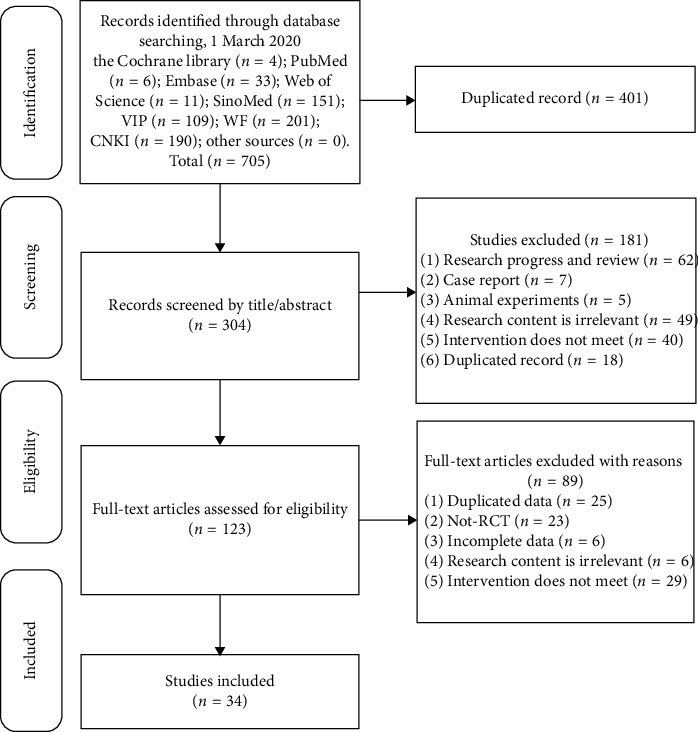
Flow chart of the study searching and selecting process.

A total of 705 studies were initially identified. After a series of procedures (removal of duplicate publications, screening of titles and abstracts, and assessment of full text), 34 [42–75] articles were finally included for analysis. The searching and selecting process was shown in Figure 1.

### 3.2. Study Characteristics

A total of 34 studies (28 two-arm studies and 6 three-arm studies [45, 47, 56–58, 62]) including 2655 patients were enrolled, among which 1344 patients were in the intervention group and 1311 patients in the control group, and 40 pairs of comparisons were provided. Among them, 21 RCTs [42–62] compared the efficacy of ACE vs. estazolam tablet (EZ), and 19 RCTs [45, 47, 56–58, 62–75] compared the efficacy of ACE vs. acupuncture (ACU). The mean age of the patients varied from 20 to 57.7 yrs in studies. The mean course of disease varied from 1.53 to 117.3 months. The duration of ACE varied from 14 to 90 days. The frequency of ACE varied from once per week to once every 30 days. Sessions of ACE varied from 1 to 36. The period of follow-up varied from 30 to 42 days. In addition, the main descriptive and clinical characteristics of these 34 studies were summarized in Supplementary file 1: Table S2.

### 3.3. Quality Assessment

**Figure 2:**
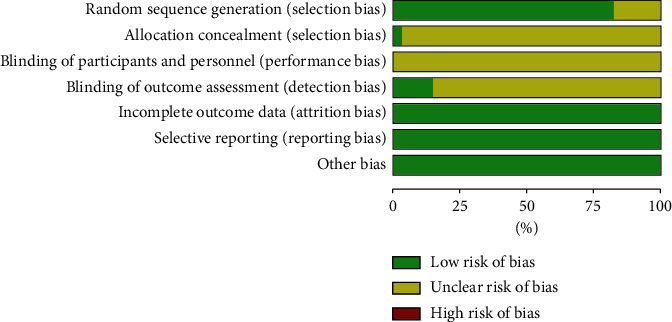
Quality assessment of included studies.

Parameters of quality assessment included the following. (a) Random sequence generation: 28 RCTs [42–45, 48–52, 54, 56–61, 63–71, 73–75] clearly described the methods of randomization. The other 6 RCTs did not specify the methods of randomization. (b) Allocation concealment: only one RCT [59] used envelopes. The other 33 RCTs were unable to be determined. (c) Blinding of participants and personnel: no study clearly illustrated or contained the allocation concealment. (d) Blinding of outcome assessment: 5 RCTs [57, 58, 63, 66, 70] mentioned blinding of outcome assessment. The other 29 RCTs were unable to be determined. (e) Incomplete outcome data: the results of 34 RCTs were complete. (f) Selective reporting and other bias: none of these 34 RCTs mentioned selective reporting and other sources of bias. The results of the quality assessment were shown in Figure 2 and Supplementary file 1: Figure S1 and Table S3.

### 3.4. Meta-Analysis

#### 3.4.1. ACE versus EZ

**Figure 3:**
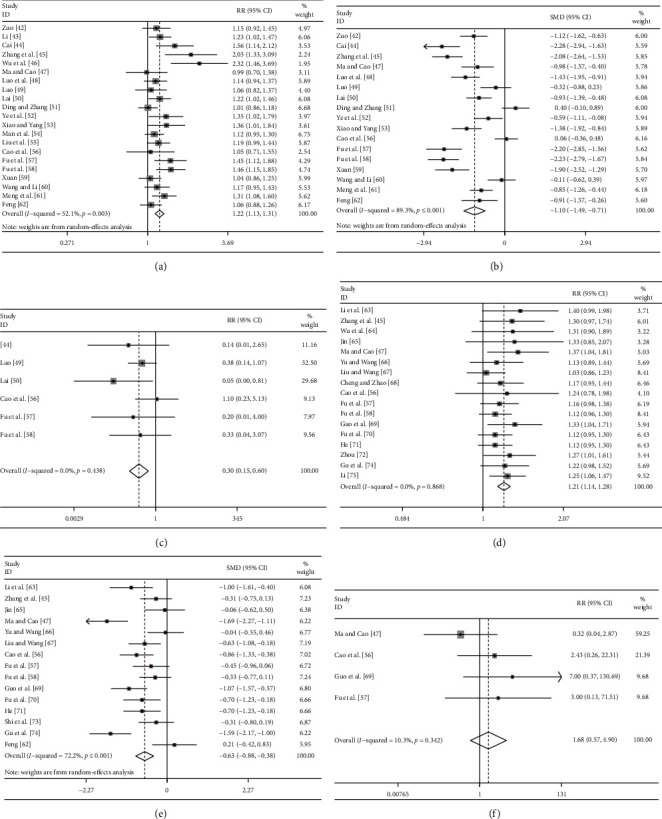
The forest plot of quantitative analysis results. ACE group versus EZ group: (a) clinical therapeutic effect; (b) PSQI score; (c) the incidence of adverse events. ACE group versus ACU group: (d) clinical therapeutic effect; (e) PSQI score; (f) the incidence of adverse events.

(1) Primary outcomes
  - *Clinical Therapeutic Effect.* 21 studies involving 1440 patients were included for analysis [42–62]. Meta-analysis showed that ACE had better efficacy in treating insomnia compared with the EZ (RR = 1.22, 95% CI: 1.13, 1.31, *P* ≤ 0.001) with a moderate heterogeneity (*P* ≤ 0.001, *I*^2^ = 52.1%) (Figure 3(a)). Six studies reported the results at a one-month follow-up [44, 45, 47, 56–58]. The result showed that ACE had a better long-term clinical efficacy compared with the EZ (RR = 1.87, 95% CI: 1.58, 2.22, *P* ≤ 0.001) with a low heterogeneity (*P*=0.38, *I*^2^ = 5.5%) (Table 1 and Supplementary file 2: Figure S1(a)).
  - *PSQI Score.* 17 studies were included for analysis [42, 44, 45, 47–53, 56–62]. The meta-analysis showed that ACE had a better effect on reducing the score of PSQI than EZ (SMD = −1.10, 95% CI: −1.49, −0.71, *P* ≤ 0.001) with high heterogeneity (*P* ≤ 0.001, *I*^2^ = 89.3%) (Figure 3(b)). Seven studies reported the results at a one-month follow-up [44, 45, 47, 49, 56–58]. Meta-analysis showed that ACE had a better long-term effect than EZ (SMD = −1.37, 95% CI: −1.87, −0.86, *P* ≤ 0.001) with high heterogeneity (*P* ≤ 0.001, *I*^*2*^ = 83.5%) (Table 1 and Supplementary file 2: Figure S2(a)).
(2) Secondary outcomes
9 studies were included for analysis of SQ, FAT, ST, SE, SD, DD, and HD scores [45, 47, 49–51, 57–59, 62]. Meta-analysis showed that ACE had a better effect on reducing the scores of SQ, SE, SD, and DD than EZ (*P* < 0.05). However, no significant effect was found on reducing the scores of FAT, ST, and HD. In addition, high heterogeneity was observed in all studies. Five studies reported the results of a one-month follow-up [45, 47, 49, 57, 58]. Meta-analysis showed that ACE had a better long-term effect on reducing the scores of SQ, FAT, ST, SE, SD, and DD than EZ (*P* < 0.05) (Table 2 and Supplementary file 2: Figures S3–S5).
(3) Adverse events
6 studies were included for analysis [44, 49, 50, 56–58]. The meta-analysis showed that the number of adverse events due to ACE was lower than that due to EZ. ACE was safer than EZ on treating insomnia (RR = 0.30, 95% CI: 0.15, 0.60, *P* ≤ 0.001) with a low heterogeneity (*P*=0.44, *I*^2^ = 0.0%) (Figure 3(c)).

#### 3.4.2. ACE versus ACU

(1) Primary outcomes
  - *Clinical Therapeutic Effect.* 17 studies involving 1111 patients were included for analysis [45, 47, 56–58, 63–72, 74, 75]. The meta-analysis showed that ACE had a better effect on treating insomnia than ACU (RR = 1.21, 95% CI: 1.14, 1.28, *P* ≤ 0.001) with a low heterogeneity (*P*=0.87, *I*^2^ = 0.0%) (Figure 3(d)). Six studies reported the results of a one-month follow-up [45, 47, 56–58, 66]. Meta-analysis showed that ACE had a better long-term clinical efficacy than ACU (RR = 1.30, 95% CI: 1.14, 1.48, *P* ≤ 0.001) with a low heterogeneity (*P*=0.64, *I*^2^ = 0.0%) (Table 1 and Supplementary file 2: Figure S1(b)).
  - *PSQI Score*. 15 studies were included for analysis [45, 47, 56–58, 62, 63, 65–67, 69–71, 73, 74]. The meta-analysis showed that ACE had a better effect on reducing the score of PSQI than ACU (SMD = −0.63, 95% CI: −0.88, −0.38, *P* ≤ 0.001) with a moderate heterogeneity (*P* ≤ 0.001, *I*^2^ = 72.2%) (Figure 3(e)). Six studies reported the results at a one-month follow-up [45, 47, 56–58, 66]. Meta-analysis showed that ACE had a better long-term effect than ACU (SMD = −0.66, 95% CI: −0.96, −0.35, *P* ≤ 0.001) with moderate heterogeneity (*P*=0.04, *I*^2^ = 56.2%) (Table 1 and Supplementary file 2: Figure S2(b)).
(2) Secondary outcomes
12 studies were included for analysis of SQ, FAT, ST, SE, SD, DD, and HD scores [45, 47, 57, 58, 62, 64, 66, 67, 69–71, 73]. Meta-analysis showed that ACE had a better effect on reducing the score of SQ, ST, SE, SD, and DD than ACU (*P* < 0.05). However, no significant effect was found on reducing the scores of FAT and HD. Five studies reported the results of a one-month follow-up [45, 47, 57, 58, 66]. Meta-analysis showed that ACE had a better long-term effect on reducing the scores of SQ, FAT, ST, SE, SD, and DD than ACU (*P* < 0.05) (Table 2 and Supplementary file 2: Figures S6–S8).
(3) Adverse events
4 studies were included for analysis [47, 56, 57, 69]. No significant difference was observed in the result (RR = 1.68, 95% CI: 0.57, 4.9, *P*=0.35 > 0.05) with low heterogeneity (*P*=0.34, *I*^2^ = 10.3%) (Figure 3(f)). It indicated that both methods were safe.

### 3.5. Sensitivity Analysis

Sensitivity analysis was used to identify the potential sources of heterogeneity and to examine the stability of the quantitative synthesis results. In the leave-one-out analysis by omitting one study each time, the overall combined results of clinical therapeutic effect (ACE versus EZ) and PSQI score (ACE versus EZ/ACU) did not change substantially. These indicated that the synthetic results were robust and no single study had a significant impact on the overall results (Supplementary file 2: Figure S9).

### 3.6. Metaregression Analysis and Subgroup Analysis

Univariate metaregression analysis and subgroup analysis were used to analyze the internal factors of these studies (mean age, mean course of the disease, duration of treatment, frequency of intervention, and the number of intervention). It was found that the above factors were not the main reason for the heterogeneity of these studies in the clinical therapeutic effect (ACE versus EZ) and PSQI score (ACE versus EZ/ACU) (all *P* values >0.05; Supplementary file 2: Figures S10–S27).

### 3.7. Publication Bias

**Figure 4:**
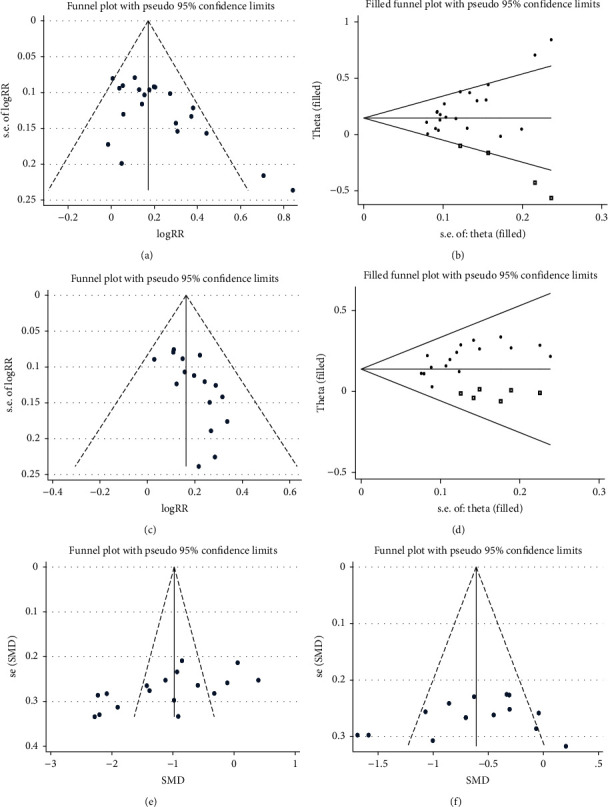
Meta-analysis results of publication bias. (a) Funnel plot with pseudo 95% confidence limits (Cls) of clinical efficacy (ACE versus EZ). The horizontal line represents the summary effect estimates, and the dotted lines are pseudo 95% Cls. (b) Filled funnel plot of RR from studies that compared the clinical efficacy between ACE and EZ. The circles alone are real studies and the circles enclosed in boxes are “filled” studies. The horizontal line represents the summary effect estimates, and the diagonal lines represent pseudo 95% Cls. Based on the random-effect model, the number of missing studies was estimated using the Linear method after 6 iterations (diff = 0), and the result was 4. (c) Funnel plot with pseudo 95% Cls of clinical efficacy (ACE versus ACU). (d) Filled funnel plot of RR from studies that compared the clinical efficacy between ACE and ACU. Based on the fixed-effect model, the number of missing studies was estimated using the Linear method after 4 iterations (diff = 0), and the result was 6. (e) Funnel plot with pseudo 95% Cls of the reduction of the PSQI score (ACE versus EZ). (f) Funnel plot with pseudo 95% Cls of the reduction of the PSQI score (ACE versus ACU).

Significant asymmetry was found upon visual inspection of the funnel plots of clinical therapeutic effect (ACE versus EZ/ACU) and the funnel plot of PSQI score (ACE versus EZ) (Figure 4). And significant publication bias was confirmed by Egger's test (all *P* values <0.05; Table 3). The sensitivity analysis using the trim-and-fill method was carried out and produced symmetrical funnel plots for them. And the corrections for potential publication bias did not alter the significant associations (Figure 4 and Table 3). It can be considered that the conclusions of the meta-analysis were robust and reliable.

### 3.8. Core Acupoints of ACE for Insomnia

**Figure 5:**
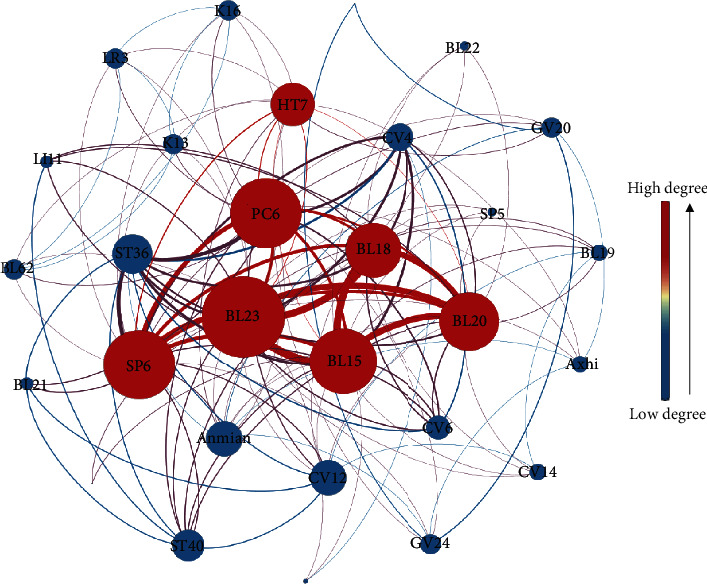
The core acupoints of ACE for insomnia analyzed by a complex network. This network contains 29 nodes and 137 edges. The higher the degree centrality value of the acupoint is, the larger the node is, the larger the font is, the redder the color of the node is, and the more important it is in the network. The thicker the edge is, the more frequently the acupoints are used together.

A total of 29 acupoints of ACE for insomnia were extracted from the 34 RCTs. Complex network analysis showed that acupoints of BL23, SP6, PC6, BL15, BL20, BL18, and HT7 were the core acupoints of ACE for insomnia according to the degree centrality and betweenness centrality (Figure 5; Supplementary file 1: Table S4). ACE was often performed once every 10 days with a duration of 1 month (Supplementary file 1: Table S2).

## 4. Discussion

As an alternative form of traditional acupuncture, acupoint catgut embedding (ACE) has advantages in the treatment of insomnia [76]. The present study has some findings. Firstly, ACE has better efficacy in managing insomnia and reducing the scores of PSQI than estazolam tablets (EZ) or acupuncture (ACU) in both short-term and long-term observations (Tables 1 and 2). Secondly, ACE is safer than EZ for insomnia. Thirdly, the core acupoints of ACE for insomnia are BL23, SP6, PC6, BL15, BL20, BL18, and HT7 (Figure 5). Lastly, ACE can reduce the visits to the clinic compared with traditional acupuncture.

Neurophysiological studies have proved that the sleep-wake circadian rhythm is maintained by the normal activities of participating structures including the central nervous system, endocrine system, and immune system [77]. A variety of neurotransmitters are involved in the regulation of the sleep-wake cycle, such as monoamines, amino acids, cytokines, choline, hormones, and skin substances [78, 79]. ACE, acupuncture, and estazolam tablets can regulate the balance of neurotransmitters, inhibit awakening and excitement, promote sleep, and regulate the rhythm of the sleep-wake cycle [80, 81]. ACE and acupuncture also can regulate sleep by regulating the content of sleep-related cytokines, which suggests that both methods can take effect through multidirectional and multitarget pathways [82]. However, it is worth noting that the use of hypnotics is not to cure insomnia but to induce patients to sleep. That is, taking hypnotics before going to bed can only help patients sleep a few hours, and when they need to sleep again, they need to take the medicine again [82]. In addition, only short-term use of benzodiazepines is recommended [83]. According to the result of adverse events (ACE versus EZ), the number of adverse events (such as pain, dizziness, nausea, dry mouth, and allergy) due to ACE was lower than that in the EZ group. This may be due to the fact that EZ is a kind of benzodiazepine. The use of benzodiazepine is always associated with higher mortality and multiple adverse events, such as drug abuse and dependence, rebound insomnia, amnesia, lung diseases, muscle relaxation, and vulnerability to fracture [83, 84]. Although ACE may also have adverse events such as low fever, subcutaneous induration, and rashes, these can be prevented or cured without special treatments [85]. Thus, ACE is superior to EZ in its efficacy for insomnia, long-term effect, and safety.

Previous studies have shown that acupuncture can regulate the sleep-wake cycle and play a sedative and hypnotic role in promoting sleep by affecting a variety of neurotransmitters, immunomodulators, hormones, skin substances, and nitric oxide [86]. Many chemicals such as 5-HT, NE, DA, GABA, Glu, IL-1, IL-6, melatonin, TNF, PGE2, and NO are closely related to the sedation and hypnosis effects of acupuncture [82]. Mechanisms of acupoint catgut embedding in the treatment of insomnia are similar to traditional acupuncture. ACE increases the contents of 5-HT, IL-1, TNF-*α,* and decreases the contents of DA and NE in the hypothalamus [81, 87]. It is found that the efficacy of ACE for insomnia is superior to traditional acupuncture and has a better long-term effect, which may be related to the therapeutic characteristics of ACE. ACE keeps stimulating acupoints with needles and catgut, which leads to a stronger and long-lasting therapeutic effect than traditional acupuncture. Therefore, it is suitable for chronic and intractable diseases such as insomnia. Catgut, an allogenic protein, is gradually softened and absorbed by the body, and this long-lasting effect can better regulate the relative balance of the internal environment and improve the regulatory ability of the body than traditional acupuncture. ACE overcomes the drawbacks of traditional acupuncture, such as short duration of therapeutic effect, difficulty in consolidating the therapeutic effect, ease to relapse, and poor patient compliance.

Acupoints used in these 34 RCTs were in different combinations, but there were seven acupoints that were the core acupoints to perform ACE for insomnia. Stimulation of BL23, SP6, PC6, BL15, BL20, BL18, and HT7 increases the expression of *γ*-aminobutyric acid and *γ*-aminobutyric acid A receptor in the hypothalamus, through which the quality of sleep is increased [88–90]. However, molecular biological mechanisms of acupoint catgut embedding in the treatment of insomnia need to be further studied due to the slight difference between ACE and ACU.

Subjective questionnaires are the main tools used to assess insomnia. Among them, PSQI is the most widely used questionnaire across different age groups in both nonclinical and clinical settings worldwide [91, 92]. PSQI has 19 individual items for the assessment of subjective sleep quality, which can be aggregated into seven components that evaluate key aspects of sleep [93]. Therefore, in the present study, the PSQI score and clinical therapeutic effect are primary outcomes. The seven components of PSQI are secondary outcomes.

### 4.1. Comparison with Previous Studies

Our findings were generally consistent with results of previous reviews written by Liu et al. [30] and Zhang et al. [31], which also showed that ACE has certain advantages in the treatment of insomnia. Besides, the present study further supplements findings in a few important respects. Firstly, previous reviews did not perform any univariate metaregression analysis and subgroup analysis. We did them to explore whether the results were affected by some confounding factors. The consistent results of sensitivity, metaregression, and subgroup analysis show that our findings are robust and reliable. Secondly, the present meta-analysis included 12 new RCTs with larger sample sizes and added about twice the number of patients of previous studies, which significantly enhanced the capability of confirming the clinical efficacy of ACE in the treatment of insomnia. Thirdly, the present study not only compares ACE and EZ but also ACE and ACU. The studies mentioned in the present paper are more comprehensive, covering more comparisons and providing more useful information through thorough statistical analyses. The present study analyzed not only the clinical therapeutic effect and the PSQI score but also the SQ, FAT, ST, SE, SD, DD, and HD scores as well as adverse events. Fourthly, the present study analyzed not only the immediate results after treatment but also the results at a one-month follow-up. More importantly, compared with previous studies, the odds ratio (OR) was modified to the risk ratio (RR) in the present study. As a result, our risk estimation is more accurate and reliable. In addition, it is worth noting that this study also explored the core acupoints to perform ACE for insomnia through complex network analysis, which showed that BL23, SP6, PC6, BL15, BL20, BL18, and HT7 are the core catgut embedding acupoints for insomnia.

### 4.2. Limitations

There are also a few limitations to this meta-analysis. Firstly, the included RCTs were mainly conducted in China reporting short- or medium-term outcomes. Therefore, there is a lack of international RCTs and long-term outcomes. Secondly, differences were found in acupoint selection and operation methods between RCTs on testing ACE, which may lead to clinical heterogeneity. For some assessments, answers to questionnaires were used to test the effective rate of ACE. This method has a strong subjectivity and lacks data on objective physiological and chemical measurements. Thirdly, there was a lack of comparison between ACE and placebo or between ACE and cognitive-behavioral therapies. These limitations may influence the reliability of our meta-analysis results.

Based on our findings, we suggest that future multicenter, large sample, randomized, double-blind, and long-term RCTs are necessary to confirm the efficacy of ACE in managing insomnia.

## 5. Conclusions

In summary, our study has three findings. (1) Acupoint catgut embedding (ACE) is more effective than estazolam tablets (EZ) or acupuncture (ACU) in the treatment of insomnia and has a better long-term efficacy based on the assessment of the pooled results (clinical therapeutic effect, reduction of PSQI, SQ, ST, SE, SD, DD, and HD scores). (2) ACE is safer than EZ for insomnia. (3) BL23, SP6, PC6, BL15, BL20, BL18, and HT7 are the core acupoints of ACE for insomnia. Considering the increasing prevalence worldwide and the advantages of enhanced efficacy and reduced visits to the clinic by ACE, the present study provides a practical and convenient alternative therapy for insomnia.

## Figures and Tables

**Table 1:** Meta-analysis results of primary outcomes. Abbreviations: *N*, number of studies; CI, confidential interval; test for heterogeneity, *I*^2^ and *P*′; RR, risk ratio; SMD, standardized mean difference; PSQI, Pittsburgh Sleep Quality Index; ACE, acupoint catgut embedding; EZ, estazolam tablets; ACU, acupuncture. *∗P*_two−tailed_ < 0.05; ^#^based on the fixed-effect model, the rest is based on the random-effect model.

Groups		Clinical efficacy RR (95% CI); heterogeneity; *N*	Reduction of PSQI score SMD (95% CI); heterogeneity; *N*
Results after treatment	ACE vs. EZ	1.22 (1.13, 1.31), *P* ≤ 0.001^*∗*^; *I*^2^ = 52.1%, *P*′≤0.001^*∗*^; *N* = 21	−1.10 (−1.49, −0.71), *P* ≤ 0.001^*∗*^; *I*^2^ = 89.3%, *P*′≤0.001^*∗*^; *N* = 17
	ACE vs. ACU	1.21 (1.14, 1.28)^#^, *P* ≤ 0.001^*∗*^; *I*^2^ = 0.0%, *P*′=0.87; *N* = 17	0.63 (−0.88, −0.38), *P* ≤ 0.001^*∗*^; *I*^2^ = 72.2%, *P*′≤0.001^*∗*^; *N* = 15
Results of a one-month follow-up	ACE vs. EZ	1.87 (1.58, 2.22)^#^, *P* ≤ 0.001^*∗*^; *I*^2^ = 5.5%, *P*′=0.38; *N* = 6	−1.37 (−1.87, −0.86), *P* ≤ 0.001^*∗*^; *I*^2^ = 83.5%, *P*′≤0.001^*∗*^; *N* = 7
	ACE vs. ACU	1.30 (1.14, 1.48)^#^, *P* ≤ 0.001^*∗*^; *I*^2^ = 0.0%, *P*′=0.64; *N* = 6;	−0.66 (−0.96, −0.35), *P* ≤ 0.001^*∗*^;*I*^2^ = 56.2%, *P*′≤0.04^*∗*^; *N* = 6

**Table 2:** Meta-analysis results of secondary outcomes. Abbreviations: SMD, standardized mean difference; CI, confidential interval; *N*, number of studies; ACE, acupoint catgut embedding; EZ, estazolam tablets; ACU, acupuncture; SQ, sleep quality; FAT, fall asleep time; ST, sleep time; SE, sleep efficiency; SD, sleep disorder; DD, daytime dysfunction; HD, hypnotic drugs; NA: unclear. *∗P*_two−tailed_ < 0.05; ^#^ based on the fixed-effect model, the rest is based on the random-effect model.

Secondary outcomes SMD (95% CI)	Results after treatment		Results of a one-month follow-up	
	ACE vs. EZ (*N* = 9)	ACE vs. ACU (*N* = 12)	ACE vs. EZ (*N* = 5)	ACE vs. ACU (*N* = 5)
Reduction of SQ score	−0.91 (−1.29, −0.53), *P* ≤ 0.001^*∗*^	−0.56 (−0.87, −0.24), *P* ≤ 0.001^*∗*^	−1.31 (−1.89, −0.73), *P* ≤ 0.001^*∗*^	−0.73 (−1.15, −0.31), *P* ≤ 0.001^*∗*^
Reduction of FAT score	−0.24 (−0.71, 0.24), *P*=0.32	−0.17 (−0.41, 0.07), *P*=0.17	−1.34 (−1.81, −0.87), *P* ≤ 0.001^*∗*^	−0.25 (−0.46, −0.03)^#^, *P*=0.03^*∗*^
Reduction of ST score	−0.04 (−0.46, 0.38), *P*=0.86	−0.29 (−0.51, −0.07), *P*=0.01^*∗*^	−0.81 (−1.04, −0.58)^#^, *P* ≤ 0.001^*∗*^	−0.50 (−0.72, −0.28)^#^, *P* ≤ 0.001^*∗*^
Reduction of SE score	−1.00 (−1.96, −0.03), *P*=0.04^*∗*^	−0.30 (−0.51, −0.09), *P*=0.01^*∗*^	−1.23 (−1.78, −0.68), *P* ≤ 0.001^*∗*^	−0.31 (−0.53, −0.10)^#^, *P*=0.01^*∗*^
Reduction of SD score	−0.51 (−0.92, −0.10), *P*=0.01^*∗*^	−0.24 (−0.39, −0.09)^#^, *P* ≤ 0.001^*∗*^	−1.13 (−1.37, −0.89)^#^, *P* ≤ 0.001^*∗*^	−0.39 (−0.61, −0.17)^#^, *P* ≤ 0.001^*∗*^
Reduction of DD score	−0.87 (−1.27, −0.46), *P* ≤ 0.001^*∗*^	−0.53 (−0.83, −0.22), *P* ≤ 0.001^*∗*^	−0.61 (−0.84, −0.39) ^#^, *P* ≤ 0.001^*∗*^	−0.30 (−0.52, −0.08)^#^, *P*=0.01^*∗*^
Reduction of HD score	−0.36 (−1.01, 0.28), *P*=0.27	−0.16 (−0.40, −0.08)^#^, *P*=0.19	NA	−0.36 (−0.74, 0.02)^#^, *P*=0.06

**Table 3:** Meta-analysis results of publication bias. Abbreviations: *N*, number of studies; CI, confidential interval; RR, risk ratio; SMD, standardized mean difference; PSQI, Pittsburgh Sleep Quality Index; ACE, acupoint catgut embedding; EZ, estazolam tablets; ACU, acupuncture. *∗P*_two−tailed_ < 0.05; ^#^ based on the fixed-effect model, the rest is based on the random-effect model.

Groups	Clinical efficacy		Reduction of PSQI score	
	Egger's test *N*; *t* value; *P* (95% CI)	Results after shear compensation RR (95% CI); *N*	Egger's test *N*; *t* value; *P* (95% CI)	Results after shear compensation SMD (95% CI); *N*
ACE vs. EZ	*N* = 21; 3.44; *P* ≤ 0.001^*∗*^ (1.15, 4.71)	1.16 (1.07, 1.25), *P* ≤ 0.001^*∗*^; *N* = 25	*N* = 17; −2.83; *P*=0.01^*∗*^ (−22.18, −3.10)	−1.10 (−1.49, −0.71), *P* ≤ 0.001^*∗*^; N=17
ACE vs. ACU	*N* = 17; 3.43; *P* ≤ 0.001^*∗*^ (0.58, 2.49)	1.15 (1.09, 1.24)^#^, *P* ≤ 0.001^*∗*^; *N* = 23	*N* = 15; −0.98; *P*=0.34 (−14.55, 5.46)	—

## Data Availability

All data used in this study are included in this article.

## Abbreviations

ACE: Acupoint catgut embedding
RCT: Clinical randomized controlled trial
EZ: Estazolam tablets
ACU: Acupuncture
CBT-I: Cognitive-behavioral therapy for insomnia
PSQI: Pittsburgh Sleep Quality Index
OR: Odds ratio
RR: Risk ratio
CCMD: The Chinese classification and diagnostic criteria of mental disorders
SQ: Sleep quality
FAT: Fall asleep time
ST: Sleep time
SE: Sleep efficiency
SD: Sleep disorder
DD: Daytime dysfunction
HD: Hypnotic drugs
CI: Confidential interval
SMD: Standardized mean difference
AA: Auricular acupressure
TMS: Transcranial magnetic stimulation
5-HT: 5-hydroxytryptamine
IL-1: Interleukin-1
IL-1: Interleukin-6
TNF: Tumor necrosis factor
GABA: *γ*-aminobutyric acid
Glu: Glutamic acid
PGE2: Prostaglandin E2
NO: Nitric oxide
TNF-*α*: Tumor necrosis factor-*α*
DA: Dopamine
NE: Norepinephrine
BL23: Shenshu
BL15: Xinshu
SP6: Sanyinjiao
BL20: Pishu
PC6: Neiguan
ST36: Zusanli
BL18: Ganshu
HT7: Shenmen
ST40: Fenglong.

## Supplementary Materials

Supplementary Materials
*Supplementary file 1.* Table S1: searching strategy for PubMed search. Table S2: basic characteristics of included studies in the present study. Table S3: quality assessment of included studies. Table S4: topological structure value of ACE acupoints for insomnia. Figure S1: quality assessment of included studies (risk of bias summary). *Supplementary file 2.* Figure S1: the forest plot of clinical therapeutic effect (one-month follow-up). Figure S2: the forest plot of reduction of PSQI score (one-month follow-up). Figures S3 and S4: forest plots of reduction of SQ, FAT, ST, SE, SD, DD, and HD scores (ACE versus EZ). Figure S5: the forest plot of reduction of SQ, FAT, ST, SE, SD, and DD scores (one-month follow-up) (ACE versus EZ). Figures S6 and S7: forest plots of reduction of SQ, FAT, ST, SE, SD, DD, and HD scores, respectively (ACE versus ACU). Figure S8: the forest plot of reduction of SQ, FAT, ST, SE, SD, and DD scores (one-month follow-up) (ACE versus ACU). Figure S9: the forest plot of sensitivity analysis. Figure S10: the graph of univariate metaregression analysis (ACE versus EZ: clinical therapeutic effect). Figures S11–S15: forest plots of subgroup analysis by mean age, mean course of disease, duration of treatment, frequency of intervention, and number of intervention, respectively (ACE versus EZ: clinical therapeutic effect). Figure S16: the graph of univariate metaregression analysis (ACE versus EZ: reduction of the PSQI score). Figures S17–S21: forest plots of subgroup analysis by mean age, mean course of disease, duration of treatment, frequency of intervention, and number of intervention, respectively (ACE versus EZ: reduction of the PSQI score). Figure S22: the graph of univariate metaregression analysis (ACE versus ACU: reduction of the PSQI score). Figures S23–S27: forest plots of subgroup analysis by mean age, mean course of disease, duration of treatment, frequency of intervention, and number of intervention, respectively (ACE versus ACU: reduction of the PSQI score).
