# Supplementary material for: Acupoint Catgut Embedding for Insomnia: A Meta-Analysis of Randomized Controlled Trials
Source: Evid Based Complement Alternat Med. 2020 Nov 6;2020:5450824. doi: 10.1155/2020/5450824 (PMC7665919; doi:10.1155/2020/5450824)
Supplement: Supplementary Materials — Supplementary file 1. Table S1: searching strategy for PubMed search. Table S2: basic characteristics of included studies in the present study. Table S3: quality assessment of included studies. Table S4: topological structure value of ACE acupoints for insomnia. Figure S1: quality assessment of included studies (risk of bias summary). Supplementary file 2. Figure S1: the forest plot of clinical therapeutic effect (one-month follow-up). Figure S2: the forest plot of reduction of PSQI score (one-month follow-up). Figures S3 and S4: forest plots of reduction of SQ, FAT, ST, SE, SD, DD, and HD scores (ACE versus EZ). Figure S5: the forest plot of reduction of SQ, FAT, ST, SE, SD, and DD scores (one-month follow-up) (ACE versus EZ). Figures S6 and S7: forest plots of reduction of SQ, FAT, ST, SE, SD, DD, and HD scores, respectively (ACE versus ACU). Figure S8: the forest plot of reduction of SQ, FAT, ST, SE, SD, and DD scores (one-month follow-up) (ACE versus ACU). Figure S9: the forest plot of sensitivity analysis. Figure S10: the graph of univariate metaregression analysis (ACE versus EZ: clinical therapeutic effect). Figures S11–S15: forest plots of subgroup analysis by mean age, mean course of disease, duration of treatment, frequency of intervention, and number of intervention, respectively (ACE versus EZ: clinical therapeutic effect). Figure S16: the graph of univariate metaregression analysis (ACE versus EZ: reduction of the PSQI score). Figures S17–S21: forest plots of subgroup analysis by mean age, mean course of disease, duration of treatment, frequency of intervention, and number of intervention, respectively (ACE versus EZ: reduction of the PSQI score). Figure S22: the graph of univariate metaregression analysis (ACE versus ACU: reduction of the PSQI score). Figures S23–S27: forest plots of subgroup analysis by mean age, mean course of disease, duration of treatment, frequency of intervention, and number of intervention, respectively (ACE versus ACU: reductio [file 5450824.f1.zip › 5450824.f1/Supplementary File 1.docx]

**Supplementary File 1**

**Table S1**. Searching strategy for PubMed search. (pp.1)

**Table S2**. Basic Characteristics of Included Studies in the present study. (pp.2-5)

**Table S3**. Quality Assessment of Included Studies. (pp.6-7)

**Table S4**. Topological structure value of ACE acupoints for insomnia. (pp.8)

**Fig S1**. Quality assessment of included studies (risk of bias summary). (pp.9)

Table S1. Searching strategy for PubMed search

| Search | Query |
| --- | --- |
| #1 | Search: ‘acupoint catgut embedding’[Title/Abstract] OR ‘catgut embedding’[Title/Abstract] OR ‘acupoint embedding’ [Title/Abstract] OR ‘acupoint therapy’[Title/Abstract] OR ‘thread-embedding acupoint’[Title/Abstract] OR ‘acupoint thread embed’[Title/Abstract] |
| #2 | Search: ‘insomnia’[Title/Abstract] OR ‘early awakening’[Title/Abstract] OR ‘sleep dysfunction’[Title/Abstract] OR ‘sleeplessness’[Title/Abstract] OR ‘agrypnia’[Title/Abstract] OR ‘sleep disorder’[Title/Abstract] |
| #3 | Search: ‘clinical trial’[Title/Abstract] OR ‘randomized controlled trial’[Title/Abstract] OR ‘randomized’[Title/Abstract] OR ‘trial’[Title/Abstract] OR ‘controlled’[Title/Abstract] OR ‘random’[Title/Abstract] OR ‘placebo’[Title/Abstract] OR ‘groups’[Title/Abstract] |
| #4 | Search: #1 and #2 and #3 |

Table S2. Basic Characteristics of Included Studies in the present Study

| Study | Diagnostic Criteria | Number T/C | Age | CD | Methods | | ACE Acupoints | Frequency | | Follow-up (max) | Sessions | Outcomes |  |
| --- | --- | --- | --- | --- | --- | --- | --- | --- | --- | --- | --- | --- | --- |
|  |  |  |  |  | T | C |  | T | C |  |  |  |  |
| Zuo XL, 2018^42^ | TCMDTS | 37/36 | 27.2 | 1.53 | ACE | EZ | BL15, BL18, BL23 | 1 per week  (4 weeks) | 1mg/qn  (4 weeks) |  | 4 | a, b |  |
| Li LD, 2018^43^ | CCMD | 40/40 | 46.9 | 3.99 | ACE | EZ | BL23,BL15,BL14 | 1 per week  (4 weeks) | 0.4mg/qn  (4 weeks) |  | 4 | a |  |
| Cai YC, 2018^44^ | CCMD | 30/30 | 35.1 | 25.1 | ACE | EZ | BL15,BL23,CV17,CV4 | 1 per week  (4 weeks) | 0.4mg/qn  (4 weeks) | 30d | 4 | a, b, j |  |
| Zhang SY,2017^45^ | CCMD | 40/39 | 40.97 | 49.86 | ACE | ACU | BL15,BL18,BL20,BL23,CV6, CV4, ST36,SP6,PC6 | 1 per 10 days (30 days) | 30min/d  (30 days) | 30d | 3 | a,b,c,d,e,f,g,h |  |
| Zhang SY1,2017^45^ | | CCMD | 40/38 | 42.01 | 50.26 | ACE | EZ | BL15,BL18,BL20,BL23,CV6, CV4, ST36,SP6,PC6 | 1 per 10 days (30 days) | 1mg/qn  (30 days) | 30d | 3 | a,b,c,d,e,f,g,h |
| Wu ZH, 2016^46^ | CCMD | 38/37 | 42.52 | 19.96 | ACE | EZ | GV20,GV24,GB20 | 1 per week  (4 weeks) | 1mg/qn  (4 weeks) |  | 4 | a |  |
| Ma Y, 2016^47^ | CCMD | 25/24 | - | - | ACE | ACU | LI11,ST40,BL23, BL18,BL20,BL15 | 1 per 10 days (30 days) | 30min/d  (30 days) | 30d | 3 | a,b,c,d,e,f,g,h,i,j |  |
| Ma Y1, 2016^47^ | CCMD | 25/26 | - | - | ACE | EZ | LI11,ST40,BL23, BL18,BL20,BL15 | 1 per 10 days (30 days) | 1mg/qn  (30 days) | 30d | 3 | a,b,c,d,e,f,g,h,i |  |
| Luo J1, 2016^48^ | CCMD | 36/36 | 41.5 | - | ACE | EZ | BL15,BL18,BL20,BL23,Anmian | 1 per 10 days (30 days) | 1mg/qn  (30 days) |  | 3 | a,b |  |
| Luo J, 2016^49^ | CCMD | 26/25 | 39.16 | 3.02 | ACE | EZ | BL15,BL18,BL20,BL23,SP10 | 1 per 2 weeks (6 weeks) | 1mg/qn  (6 weeks) | 42d | 3 | a,b,c,d,e,g,h,j |  |

Table S2. Basic Characteristics of Included Studies in the present Study (Continued)

| Lai QH, 2015^50^ | CCMD | 45/37 | 48.68 | 14.4 | ACE | EZ | ST36,SP6,Anmian | 1 per 2 weeks (4 weeks) | 1mg/qn  (4 weeks) |  | 2 | a,b,c,d,e,f,g,h,j |
| --- | --- | --- | --- | --- | --- | --- | --- | --- | --- | --- | --- | --- |
| Ding C, 2015^51^ | CCMD | 33/31 | 42.55 | 19.97 | ACE | EZ | GV20,GV24,GB20 | 1 per week  (4 weeks) | 1mg/qn  (4 weeks) |  | 4 | a,b,c,d,e,f,g,h |
| Ye CH, 2014^52^ | CCMD | 30/30 | 47.48 | 41.63 | ACE | EZ | BL15,BL14,BL23 | 1 per week  (4 weeks) | 0.4mg/qn  (4 weeks) |  | 4 | a,b |
| Xiao YC, 2014^53^ | CCMD | 36/30 | 57.74 | - | ACE | EZ | BL15,BL18,BL20,BL23 | 1 per week  (4 weeks) | 1mg/qn  (4 weeks) |  | 4 | a,b |
| Man XL, 2014^54^ | CCMD | 30/30 | 34.5 | 60 | ACE | EZ | PC6 | 3 per week  (12 weeks) | 1mg/qn  (12 weeks) |  | 36 | a |
| Liu ZX, 2014^55^ | CCMD | 40/40 | 49.7 | 5.45 | ACE | EZ | BL23,KI3,LR3, SP6,BL62,KI6, HT7,PC6 | 1 per week  (4 weeks) | 2mg/qn  (4 weeks) |  | 4 | a |
| Cao XL, 2014^56^ | CCMD | 42/34 | 55.11 | - | ACE | ACU | BL15,BL18,BL20,BL23,SP6 | 1 per 10 days (30 days) | 30min/d  (30 days) | 30d | 3 | a,b,j |
| Cao XL1, 2014^56^ | CCMD | 42/46 | 55.9 | - | ACE | EZ | BL15,BL18,BL20,BL23,SP6 | 1 per 10 days (30 days) | 1mg/qn  (30 days) | 30d | 3 | a,b,j |
| Xu F1, 2013^57^ | CCMD | 40/40 | 43.79 | 20.38 | ACE | ACU | BL15,BL18,BL23,CV4,PC6,ST36, SP6 | 1 per 10 days (30 days) | 30min/d  (30 days) | 30d | 3 | a,b,c,d,e,f,g,h,j |
| Xu F2, 2013^57^ | CCMD | 40/40 | 43.02 | 22.27 | ACE | EZ | BL15,BL18,BL23,CV4,PC6,ST36, SP6 | 1 per 10 days (30 days) | 1mg/qn  (30 days) | 30d | 3 | a,b,c,d,e,f,g,h,j |
| Xu F, 2013^58^ | CCMD | 30/30 | 41.77 | 20.32 | ACE | ACU | BL21,ST40,CV12,SP6,PC6,ST36 | 1 per 10 days (30 days) | 30min/d  (30 days) | 30d | 3 | a,b,c,d,e,f,g,h,j |
| Xu F0, 2013^58^ | CCMD | 30/30 | 41.37 | 22.01 | ACE | EZ | BL21,ST40,CV12,SP6,PC6,ST36 | 1 per 10 days (30 days) | 1mg/qn  (30 days) | 30d | 3 | a,b,c,d,e,f,g,h,j |

Table S2. Basic Characteristics of Included Studies in the present Study (Continued)

| Huang YX,2013^59^ | CCMD | 30/30 | 39.72 | 5.64 | ACE | EZ | BL15,BL18 | 1 per 2 weeks (6 weeks) | 1mg/qn  (6 weeks) |  | | 3 | a,b,c,d,e,f,g,h,i |
| --- | --- | --- | --- | --- | --- | --- | --- | --- | --- | --- | --- | --- | --- |
| Wang XB,2012^60^ | CCMD | 30/30 | 48 | 13.48 | ACE | EZ | BL20,BL18,SP6, BL23,BL15 | 1 per 2 weeks (4 weeks) | 1mg/qn  (4 weeks) |  | | 2 | a,b,j |
| Meng S, 2011^61^ | TCMDTS | 50/50 | 46 | 58.2 | ACE | EZ | BL15,BL18,BL20,BL23,BL13 | 1 per week  (4 weeks) | 2mg/qn  (4 weeks) |  | | 4 | a,b |
| Feng QY, 2009^62^ | CCMD | 20/20 | 43.65 | 60.48 | ACE | ACU | BL15,BL18,BL20,BL23 | 1 per 15 days (30 days) | 30min/d  (30 days) |  | | 2 | b,c,d,e,f,g,h,i |
| Feng QY, 2009^62^ | CCMD | 20/20 | 44.53 | 61.36 | ACE | EZ | BL15,BL18,BL20,BL23 | 1 per 15 days (30 days) | 1mg/qn  (30 days) |  | | 2 | a,b,c,d,e,f,g,h,i |
| Li HW, 2018^63^ | CCMD | 24/24 | 56.1 | 28.02 | ACE | ACU | HT7,BL15,BL20, BL23,SP6 | 1 per 2 weeks (4 weeks) | 30min/d  (4 weeks) |  | | 2 | a,b |
| Wu J, 2016^64^ | TCMDTS | 20/20 | 36.4 | - | ACE | ACU | BL15,BL23,BL18,BL20,BL19 | 1 per 2 weeks (2 weeks) | 30min/3 per week (2 weeks) | |  | 1 | a,c,d,e,f |
| Jin J,  2016^65^ | CCMD | 30/32 | - | - | ACE | ACU | PC6 | 1 per 10 days (30 days) | 30min/d  (30 days) |  | | 3 | a,b |
| Yu XJ, 2015^66^ | CCMD | 30/30 | 49 | 9.59 | ACE | ACU | BL15,BL18,BL20,BL23,CV6, CV4, PC6,ST36,SP6 | 1 per week  (3 weeks) | 30min/d  (3 weeks) | 30d | | 3 | a,b,c,d,e,f,g,h,i |
| Liu ZL, 2015^67^ | CCMD | 40/40 | 50 | 117.3 | ACE | ACU | Ashi | 1 per 2 weeks (12 weeks) | 30min/d  (12 weeks) |  | | 6 | a,b,c,d,e,f,g,h |
| Cheng H, 2014^68^ | CCMD | 35/32 | 43 | 13.58 | ACE | ACU | PC6,HT7,SP6,GV24,GV20, Anmian | 1 per 30 days (90 days) | 30min/d  (90 days) |  | | 3 | a |
| Guo AS, 2013^69^ | CCMD | 35/35 | 20 | 46.8 | ACE | ACU | HT7,SP6,GV20, Anmian | 1 per 2 weeks (6 weeks) | 30min/d  (6 weeks) |  | | 3 | a,b,c,d,e,f,g,h,j |

Table S2. Basic Characteristics of Included Studies in the present Study (Continued)

| Xu F, 2012^70^ | CCMD | 30/30 | 49.19 | 66.85 | ACE | ACU | BL15,SP6,BL20, PC6,ST36 | 1 per 10 days (30 days) | 30min/d  (30 days) | |  | 3 | a,b,c,d,e,f,g,h,i |
| --- | --- | --- | --- | --- | --- | --- | --- | --- | --- | --- | --- | --- | --- |
| He XP, 2012^71^ | CCMD | 30/30 | 49.2 | 66.85 | ACE | ACU | BL15,SP6,BL20, PC6,ST36 | 1 per 10 days (30 days) | 30min/d  (30 days) | |  | 3 | a,b,c,d,e,f,g,h,i |
| Zhou WA,2011^72^ | CCMD | 30/30 | - | - | ACE | ACU | BL15,BL23 | 1 per week  (8 weeks) | 30min/3 per week (8 weeks) |  | | 8 | a |
| Shi YJ, 2011^73^ | CCMD | 33/31 | 41.99 | 19.48 | ACE | ACU | GV20,GV24, GB20 | 1 per week  (4 weeks) | 30min/3 per week (4 weeks) |  | | 4 | b,c,d,e,f,g,h |
| Gu RX, 2011^74^ | CCMD | 30/30 | 44.5 | 39.18 | ACE | ACU | BL15,BL20,BL18,BL19,BL23,  CV14,CV12 | 1 per 15 days (60 days) | 30min/1 per 2 days  (60 days) | |  | 4 | a,b |
| Li ZP, 2006^75^ | TCMDTS | 52/48 | 42.38 | 81.02 | ACE | ACU | BL15,BL18,BL20,BL23 | 1 per 15 days (60 days) | 30min/d  (60 days) | |  | 4 | a |

Notes: Study: First Author, year; Age: mean age (year); CD: mean course of disease (month); T: Treatment Group; C: Control Group; ACE: acupoint catgut embedding; Sessions: sessions of ACE; CCMD: the Chinese classification and diagnostic criteria of mental disorders^35^; TCMDTS: Standard for Diagnosis and Therapeutic Effect of Diseases and Syndromes in Traditional Chinese Medicine^34^; ICD: international Classification of Diseases^33^; EZ: Estazolam tablets; ACU: acupuncture; qn: quaque nocte; bid: twice a day; tid: three times a day; a: clinical therapeutic effect; b: PSQI score; c: sleep quality score; d: sleep time score; e: fall asleep time score; f: sleep efficiency score; g: sleep disorder score; h: daytime dysfunction score; i: hypnotic drugs score; j: adverse events.

Table S3. Quality Assessment of Included Studies

| **Reference number** |  | **First Author, year** | **Random Method** | **Allocation Concealment** | **Blinding of Participants and Personnel** | **Blinding of Outcome Assessment** | **Incomplete Outcome Data** | **Selective Reporting** | **Other Bias** |
| --- | --- | --- | --- | --- | --- | --- | --- | --- | --- |
| 42 |  | Zuo XL,2018 | L (random number table) | U | U | U | L | L | L |
| 43 |  | Li LD,2018 | L (random number table) | U | U | U | L | L | L |
| 44 |  | Cai YC,2018 | L (random number table) | U | U | U | L | L | L |
| 45 |  | Zhang SY,2017 | L (random number table) | U | U | U | L | L | L |
| 46 |  | Wu ZH,2016 | U | U | U | U | L | L | L |
| 47 |  | Ma Y,2016 | U | U | U | U | L | L | L |
| 48 |  | Luo J1,2016 | L (random number table) | U | U | U | L | L | L |
| 49 |  | Luo J,2016 | L (random number table) | U | U | U | L | L | L |
| 50 |  | Lai QH,2015 | L (treatment sequence) | U | U | U | L | L | L |
| 51 |  | Ding C,2015 | L (random number table) | U | U | U | L | L | L |
| 52 |  | Ye CH,2014 | L (treatment sequence) | U | U | U | L | L | L |
| 53 |  | Shao YC,2014 | U | U | U | U | L | L | L |
| 54 |  | Man XL,2014 | L (random number table) | U | U | U | L | L | L |
| 55 |  | Liu ZX,2014 | U | U | U | U | L | L | L |
| 56 |  | Cao XL,2014 | L (random number table) | U | U | U | L | L | L |
| 57 |  | Xu F1 | L (random number table) | U | U | Single blinding | L | L | L |
| 58 |  | Xu F,2013 | L (random number table) | U | U | Single blinding | L | L | L |
| 59 |  | Huang YX,2013 | L (random number table) | L(Envelope) | U | U | L | L | L |
| 60 |  | Wang XB,2012 | L (random number table) | U | U | U | L | L | L |
| 61 |  | Meng S,2011 | L (treatment sequence) | U | U | U | L | L | L |
| 62 |  | Feng QY,2009 | U | U | U | U | L | L | L |

Table S3. Quality Assessment of Included Studies (Continued)

| 63 | Li HW,2018 | L (random number table) | U | U | Single blinding | L | L | L |
| --- | --- | --- | --- | --- | --- | --- | --- | --- |
| 64 | Wu J,2016 | L (random number table) | U | U | U | L | L | L |
| 65 | Jin J,2016 | L (treatment sequence) | U | U | U | L | L | L |
| 66 | Yu XJ,2015 | L (random number table) | U | U | Single blinding | L | L | L |
| 67 | Liu ZL,2015 | L (random number table) | U | U | U | L | L | L |
| 68 | Cheng H,2014 | L (treatment sequence) | U | U | U | L | L | L |
| 69 | Guo AS,2013 | L (treatment sequence) | U | U | U | L | L | L |
| 70 | Xu F,2012 | L (random number table) | U | U | Single blinding | L | L | L |
| 71 | He XP,2012 | L (random number table) | U | U | U | L | L | L |
| 72 | Zhou WA,2011 | U | U | U | U | L | L | L |
| 73 | Shi YJ,2011 | L (random number table) | U | U | U | L | L | L |
| 74 | Gu RX,2011 | L (treatment sequence) | U | U | U | L | L | L |
| 75 | Li ZP,2006 | L (treatment sequence) | U | U | U | L | L | L |

Notes: L: Low risk; H: High risk; U: Unclear risk.

Table S4. Topological structure value of ACE acupoints for insomnia.

| **label** | **Degree**  **centrality** | **Betweenness centrality** | **label** | **Degree centrality** | **Betweenness centrality** |
| --- | --- | --- | --- | --- | --- |
| BL23 | 23 | 78.78 | LR3 | 7 | 0 |
| SP6 | 20 | 47.27 | KI3 | 7 | 0 |
| PC6 | 20 | 47.27 | KI6 | 7 | 0 |
| BL15 | 19 | 39.78 | BL62 | 7 | 0 |
| BL20 | 17 | 20.30 | BL19 | 6 | 0 |
| BL18 | 16 | 16.26 | CV14 | 6 | 0 |
| HT7 | 13 | 16.85 | Ashi | 6 | 0 |
| ST36 | 12 | 3.77 | LI11 | 5 | 0 |
| Anmian | 11 | 12.76 | BL21 | 5 | 0 |
| CV12 | 11 | 6.10 | BL13 | 4 | 0 |
| ST40 | 10 | 3.82 | SP10 | 4 | 0 |
| CV4 | 9 | 3.03 | CV17 | 3 | 0 |
| CV6 | 8 | 0 | BL14 | 2 | 0 |
| GV20 | 7 | 13 | GB20 | 2 | 0 |
| GV24 | 7 | 13 | - | - | - |

**
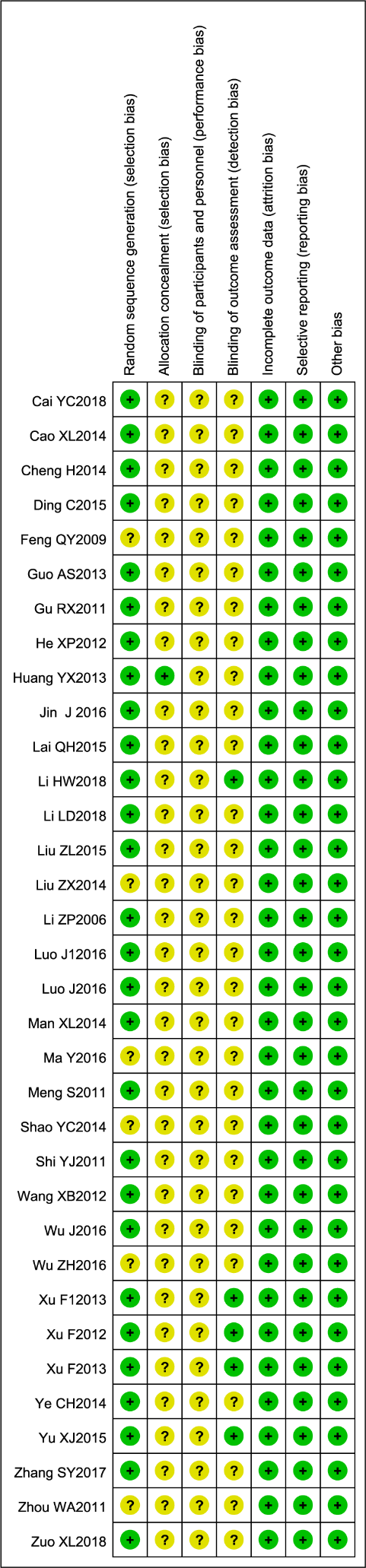
**

Figure S1 Quality assessment of included studies (risk of bias summary)
