# Supplementary material for: Acupoint Catgut Embedding for Insomnia: A Meta-Analysis of Randomized Controlled Trials
Source: Evid Based Complement Alternat Med. 2020 Nov 6;2020:5450824. doi: 10.1155/2020/5450824 (PMC7665919; doi:10.1155/2020/5450824)
Supplement: Supplementary Materials — Supplementary file 1. Table S1: searching strategy for PubMed search. Table S2: basic characteristics of included studies in the present study. Table S3: quality assessment of included studies. Table S4: topological structure value of ACE acupoints for insomnia. Figure S1: quality assessment of included studies (risk of bias summary). Supplementary file 2. Figure S1: the forest plot of clinical therapeutic effect (one-month follow-up). Figure S2: the forest plot of reduction of PSQI score (one-month follow-up). Figures S3 and S4: forest plots of reduction of SQ, FAT, ST, SE, SD, DD, and HD scores (ACE versus EZ). Figure S5: the forest plot of reduction of SQ, FAT, ST, SE, SD, and DD scores (one-month follow-up) (ACE versus EZ). Figures S6 and S7: forest plots of reduction of SQ, FAT, ST, SE, SD, DD, and HD scores, respectively (ACE versus ACU). Figure S8: the forest plot of reduction of SQ, FAT, ST, SE, SD, and DD scores (one-month follow-up) (ACE versus ACU). Figure S9: the forest plot of sensitivity analysis. Figure S10: the graph of univariate metaregression analysis (ACE versus EZ: clinical therapeutic effect). Figures S11–S15: forest plots of subgroup analysis by mean age, mean course of disease, duration of treatment, frequency of intervention, and number of intervention, respectively (ACE versus EZ: clinical therapeutic effect). Figure S16: the graph of univariate metaregression analysis (ACE versus EZ: reduction of the PSQI score). Figures S17–S21: forest plots of subgroup analysis by mean age, mean course of disease, duration of treatment, frequency of intervention, and number of intervention, respectively (ACE versus EZ: reduction of the PSQI score). Figure S22: the graph of univariate metaregression analysis (ACE versus ACU: reduction of the PSQI score). Figures S23–S27: forest plots of subgroup analysis by mean age, mean course of disease, duration of treatment, frequency of intervention, and number of intervention, respectively (ACE versus ACU: reductio [file 5450824.f1.zip › 5450824.f1/Supplementary File 2.docx]

**Supplementary Information File 2**

**Fig S1**. The forest plot of clinical therapeutic effect (one-month follow-up). (pp.2)

**Figure S2**. The forest plot of reduction of PSQI score (one-month follow-up). (pp.3)

**Fig S3** and **Fig S4**. Forest plots of reduction of SQ, FAT, ST, SE, SD, DD, and HD scores (ACE versus EZ). (pp.4-5)

**Fig S5**. The forest plot of reduction of SQ, FAT, ST, SE, SD, and DD scores (one-month follow-up) (ACE versus EZ). (pp.6)

**Fig S6**. and **Fig S7**. Forest plots of reduction of SQ, FAT, ST, SE, SD, DD, and HD scores, respectively (ACE versus ACU). (pp.7-8)

**Fig S8**. The forest plot of reduction of SQ, FAT, ST, SE, SD and DD scores (one-month follow-up) (ACE versus ACU). (pp.9)

**Fig S9**. The forest plot of sensitivity analysis. (pp.10)

**Fig** **S10**. The graph of univariate meta regression analysis (ACE versus EZ: clinical therapeutic effect). (pp.11)

**Fig S11** to **Fig S15**. Forest plots of subgroup analysis by mean age, mean course of disease, duration of treatment, frequency of intervention, and number of intervention, respectively (ACE versus EZ: clinical therapeutic effect). (pp.12-16)

**Fig S16**. The graph of univariate meta regression analysis (ACE versus EZ: reduction of the PSQI score). (pp.17)

**Fig S17** to **Fig S21**. Forest plots of subgroup analysis by mean age, mean course of disease, duration of treatment, frequency of intervention, and number of intervention, respectively (ACE versus EZ: reduction of the PSQI score). (pp.18-22)

**Fig S22**. The graph of univariate meta regression analysis (ACE versus ACU: reduction of the PSQI score). (pp.23)

**Fig S23** to **Fig S27**. Forest plots of subgroup analysis by mean age, mean course of disease, duration of treatment, frequency of intervention, and number of intervention, respectively (ACE versus ACU: reduction of the PSQI score). (pp.24-28)


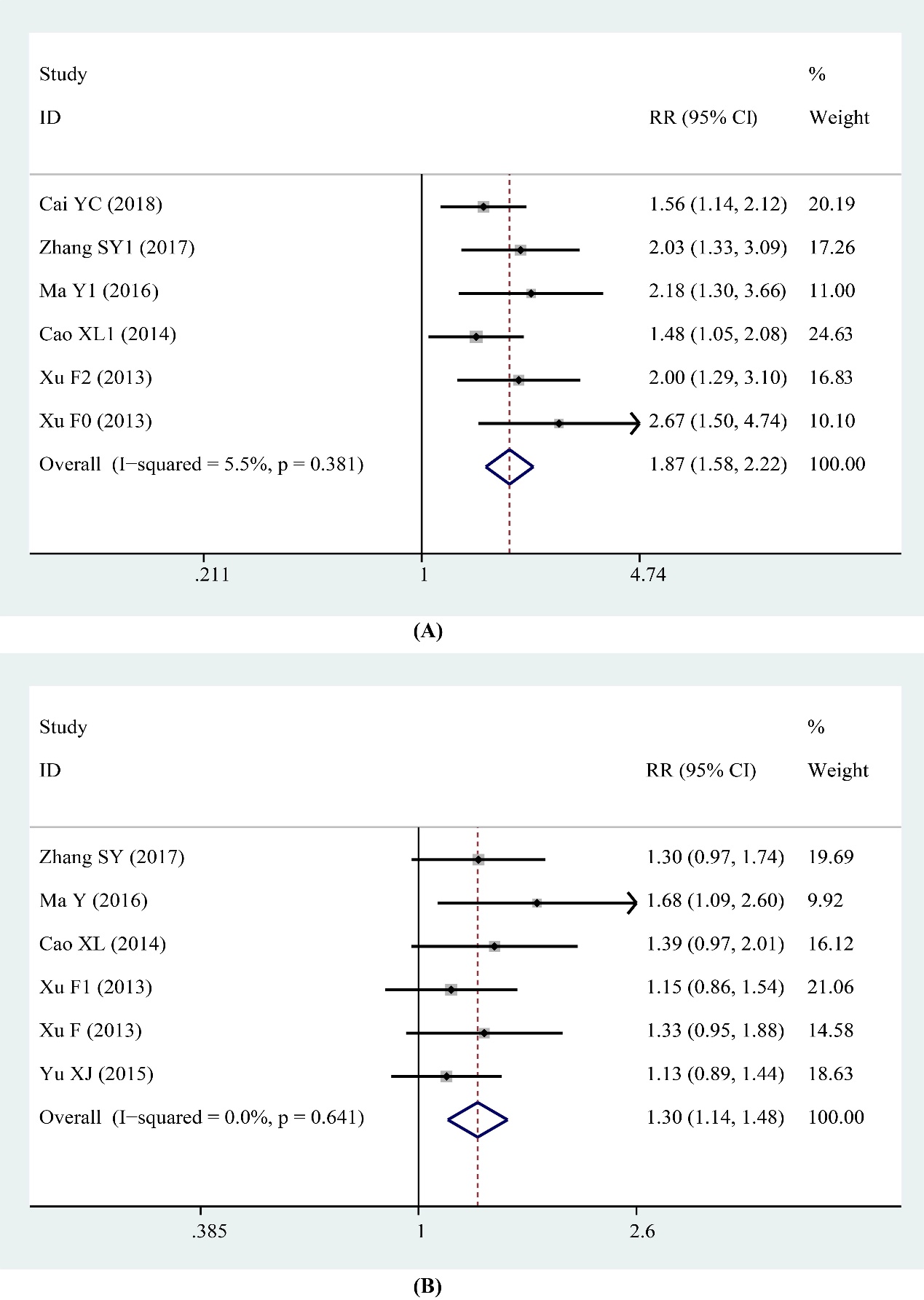


**Fig S1**. The forest plot of clinical therapeutic effect (one-month follow-up). (A): ACE group versus EZ group; (B): ACE group versus ACU group.


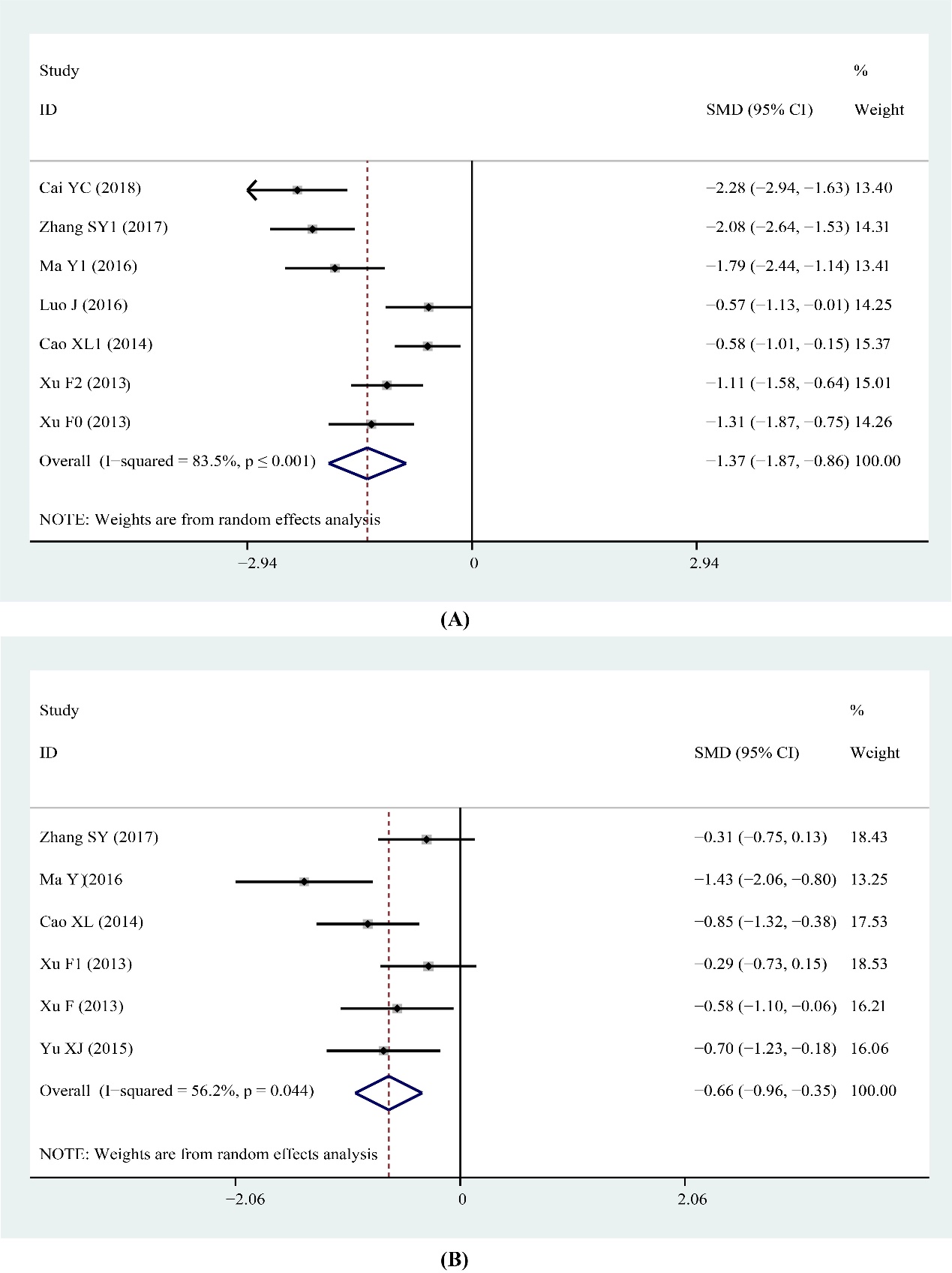


**Fig S2**. The forest plot of reduction of the PSQI score (one-month follow-up). (A): ACE group versus EZ group; (B): ACE group versus ACU group.


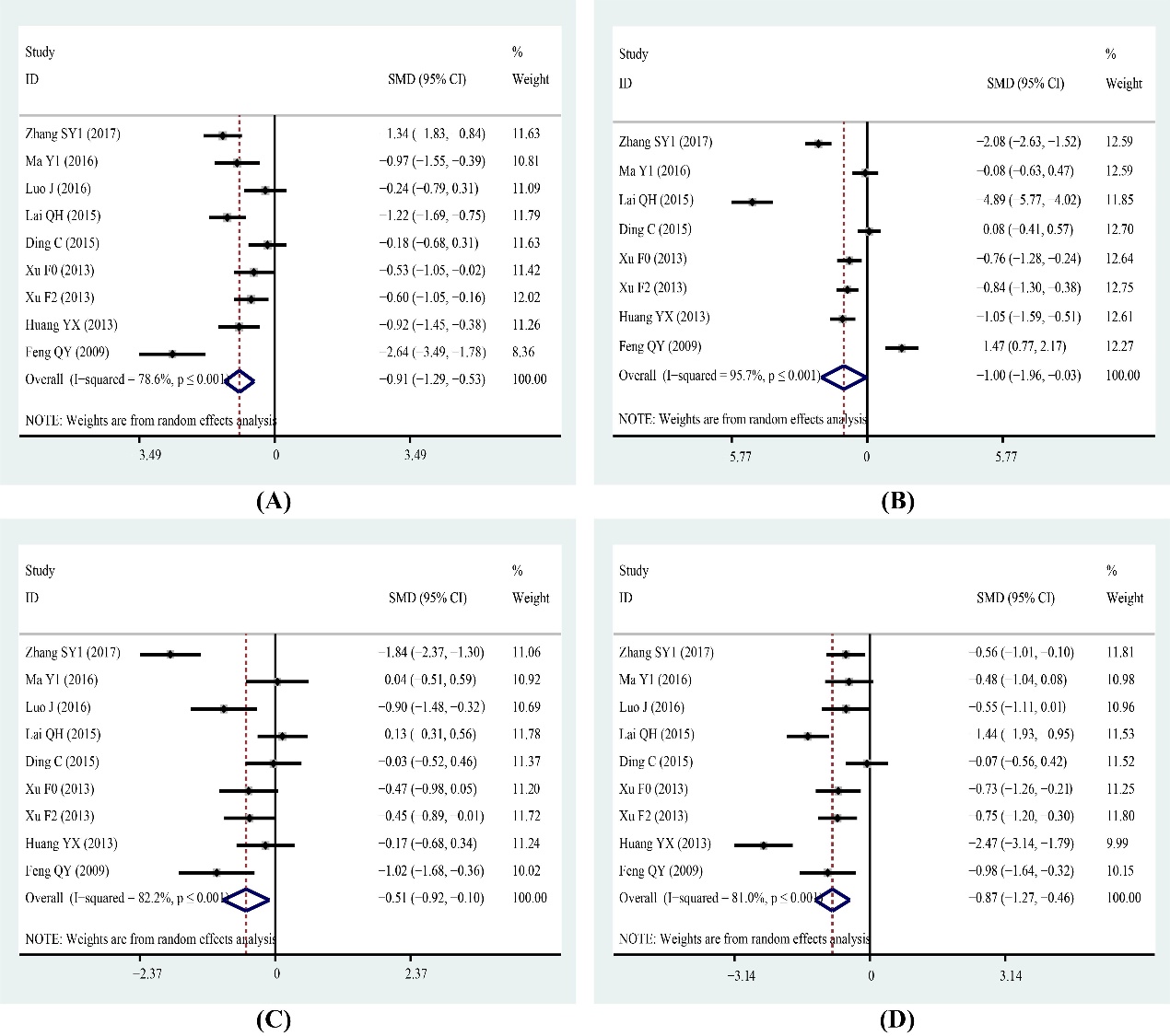


**Fig** **S3**. The forest plot of reduction of the SQ, SE, SD, and DD scores (ACE group versus EZ group). (A): reduction of the SQ score; (B): reduction of the SE score; (C) reduction of the SD score; (D) reduction of the DD score.


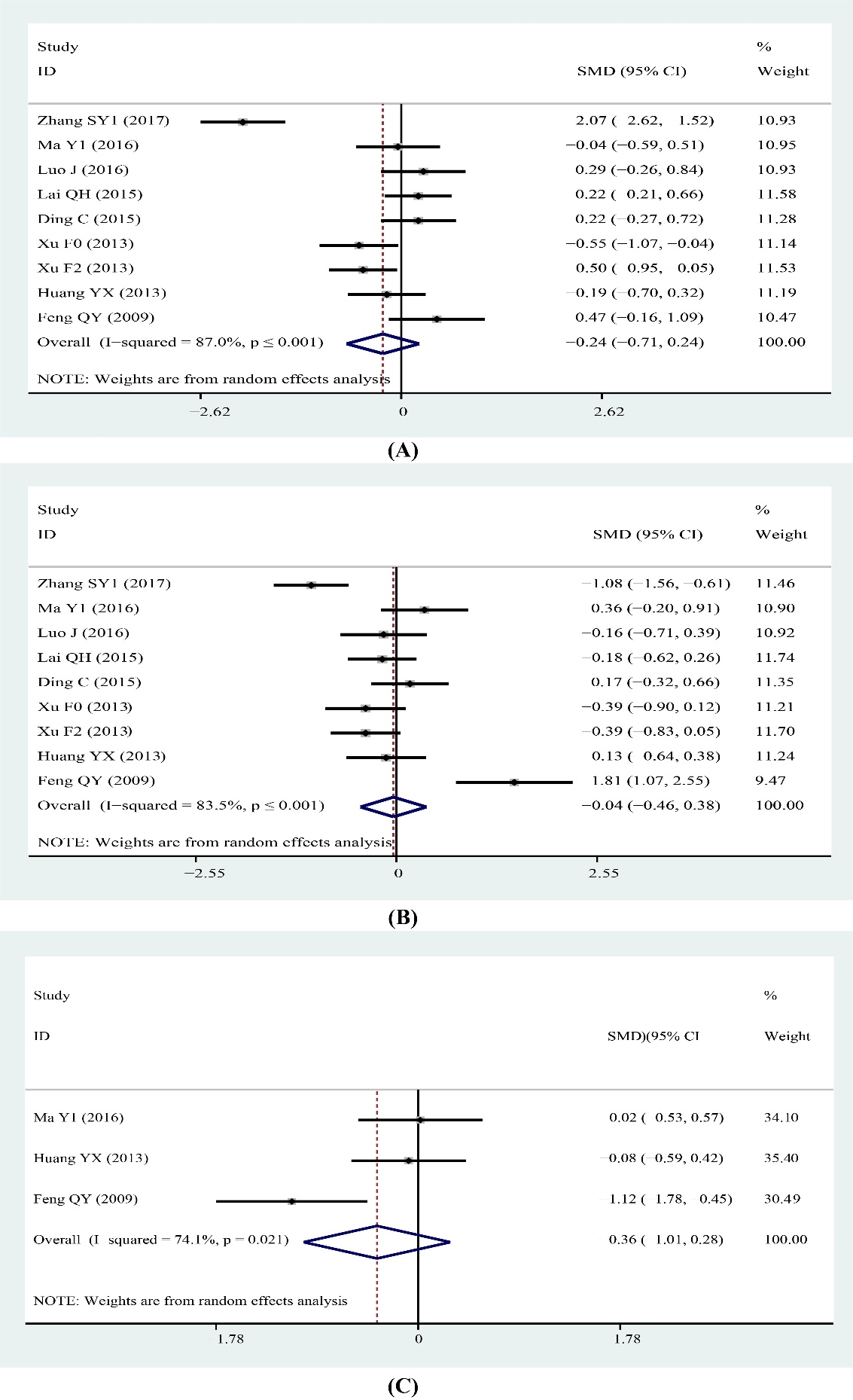


**Fig** **S4**. The forest plot of reduction of the FAT, ST, and HD scores (ACE group versus EZ group). (A): reduction of the FAT score; (B): reduction of the ST score; (C): reduction of the HD score.


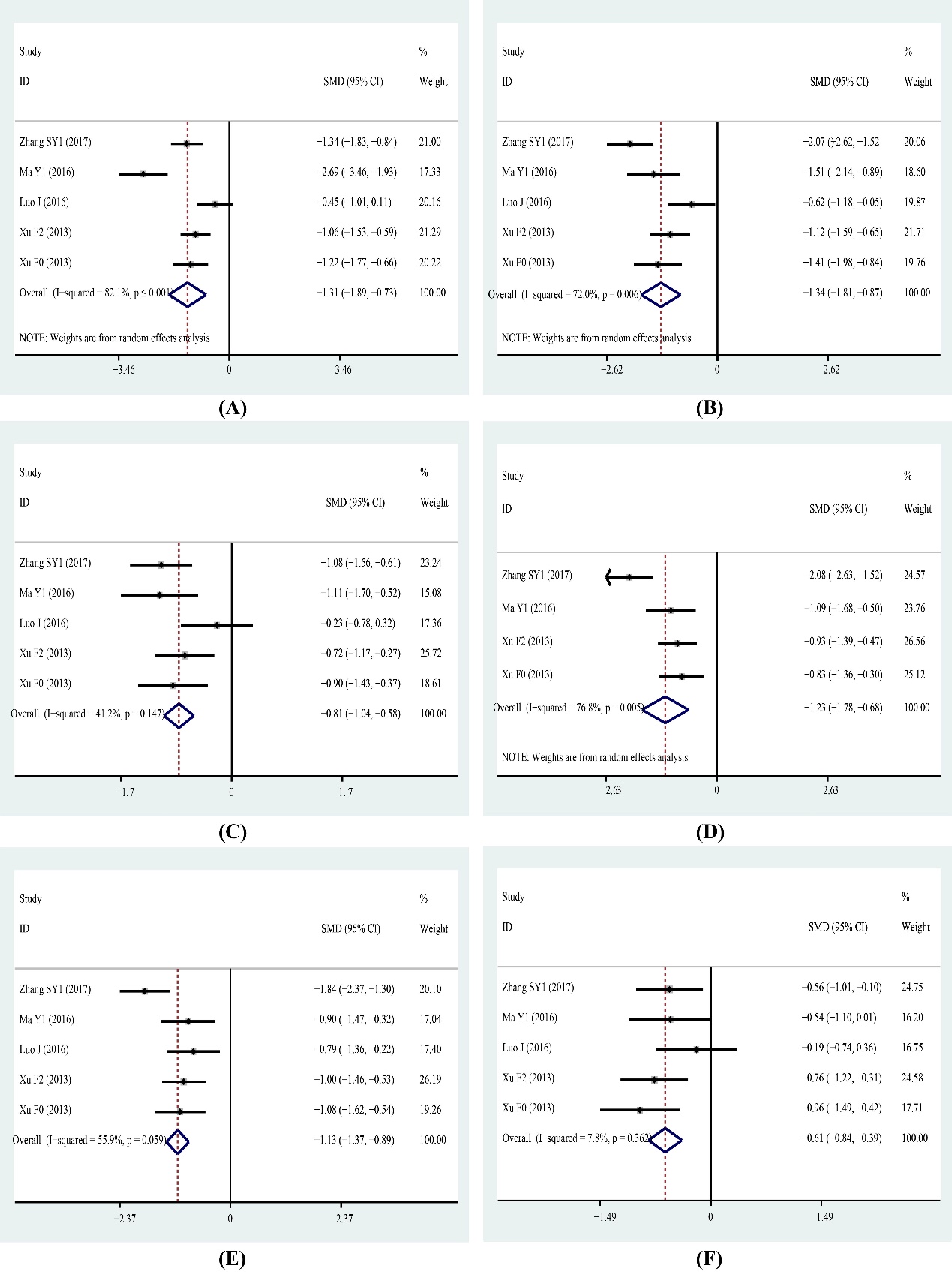


**Fig** **S5**. The forest plot of reduction of the SQ, FAT, ST, SE, SD, and DD scores (one-month follow-up) (ACE group versus EZ group). (A): reduction of the SQ score; (B): reduction of the FAT score; (C) reduction of the ST score; (D) reduction of the SE score; (D) reduction of the SD score; (F) reduction of the DD score.


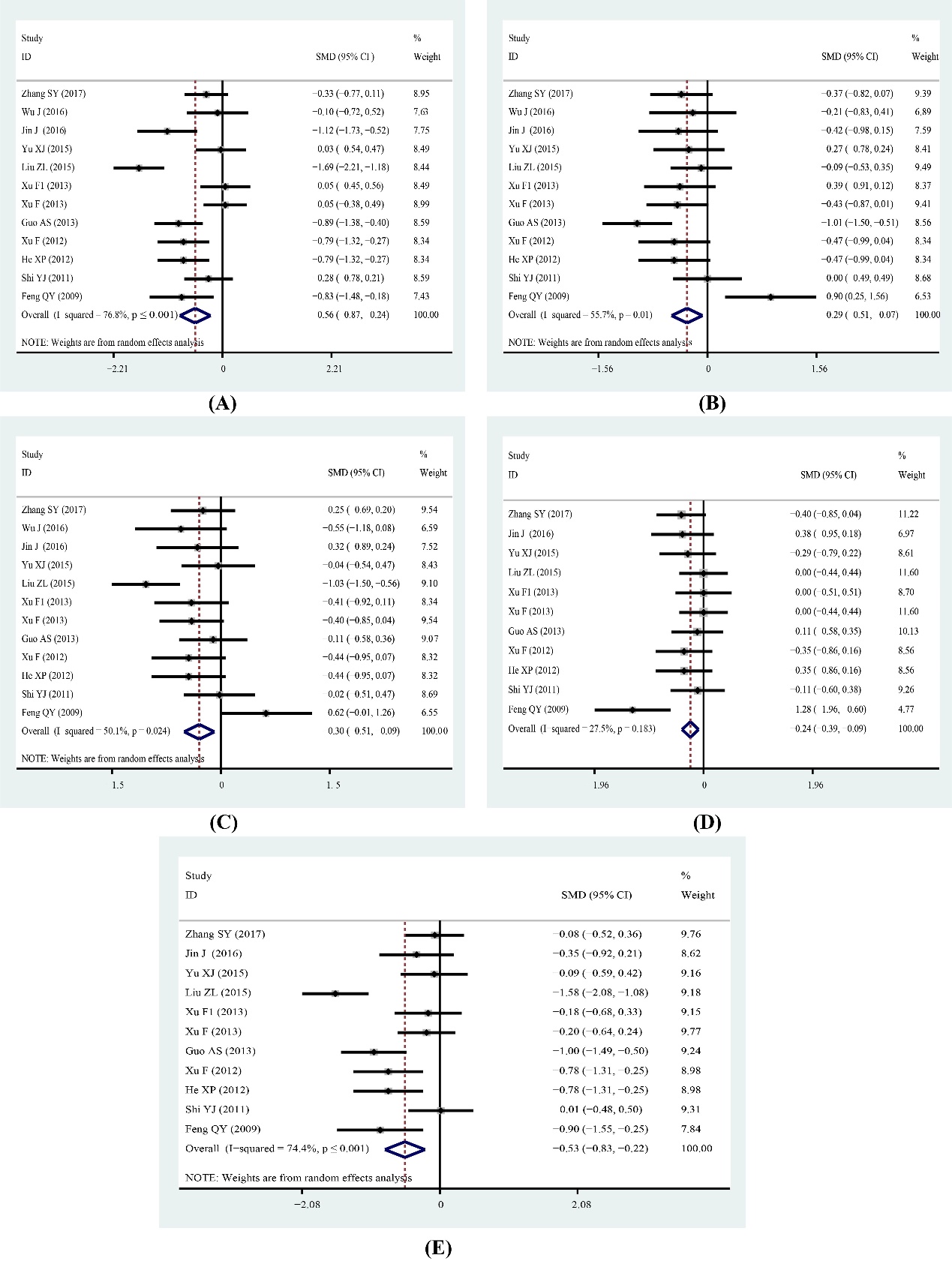


**Fig S6**. The forest plot of reduction of the SQ, ST, SE, SD, and DD scores (ACE group versus ACU group). (A): reduction of the SQ score; (B): reduction of the ST score; (C) reduction of the SE score; (D) reduction of the SD score; (E) reduction of the DD score.


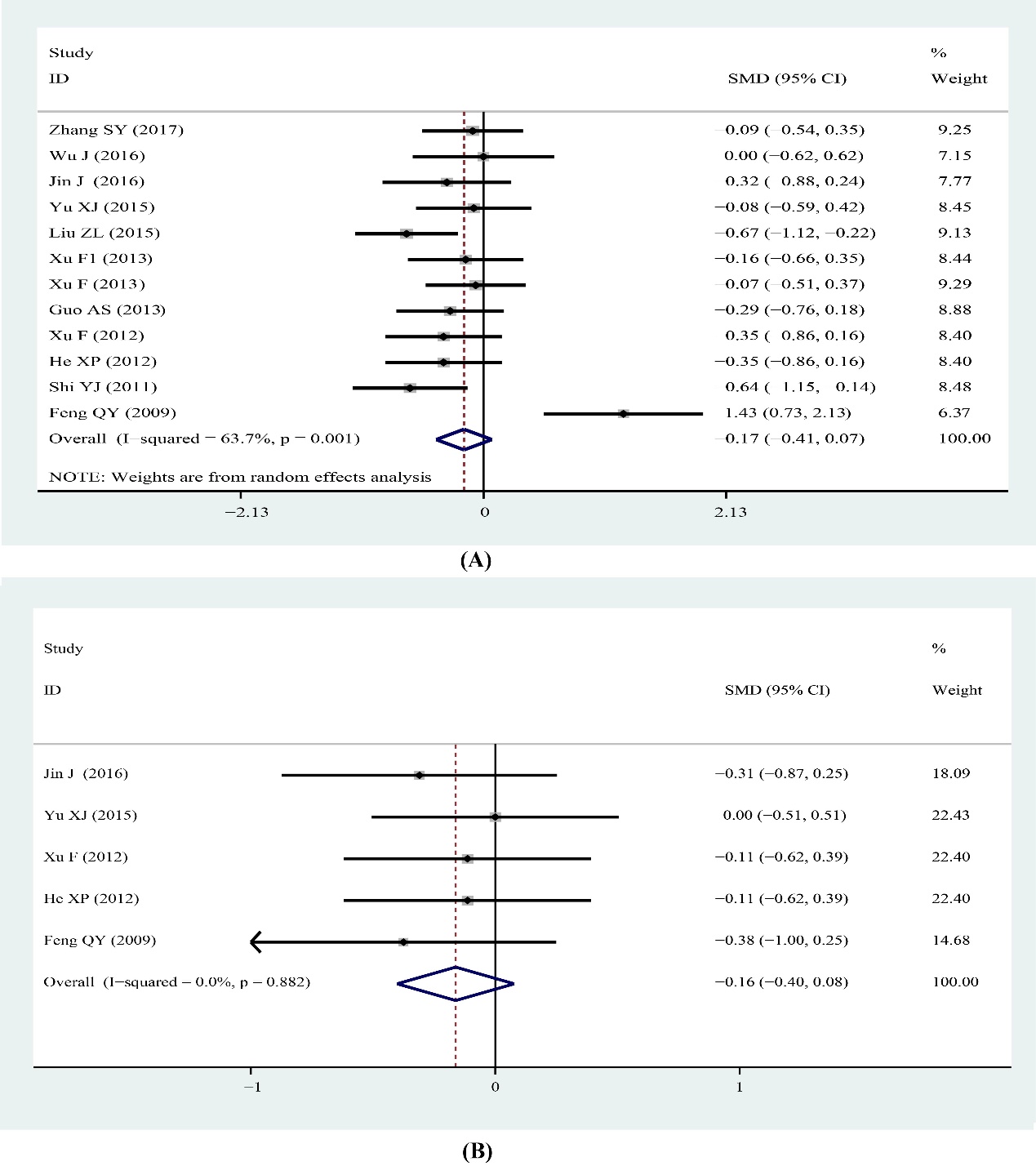


**Fig** **S7**. The forest plot of reduction of the FAT and HD scores (ACE group versus ACU group). (A): reduction of the FAT score; (B): reduction of the HD score.


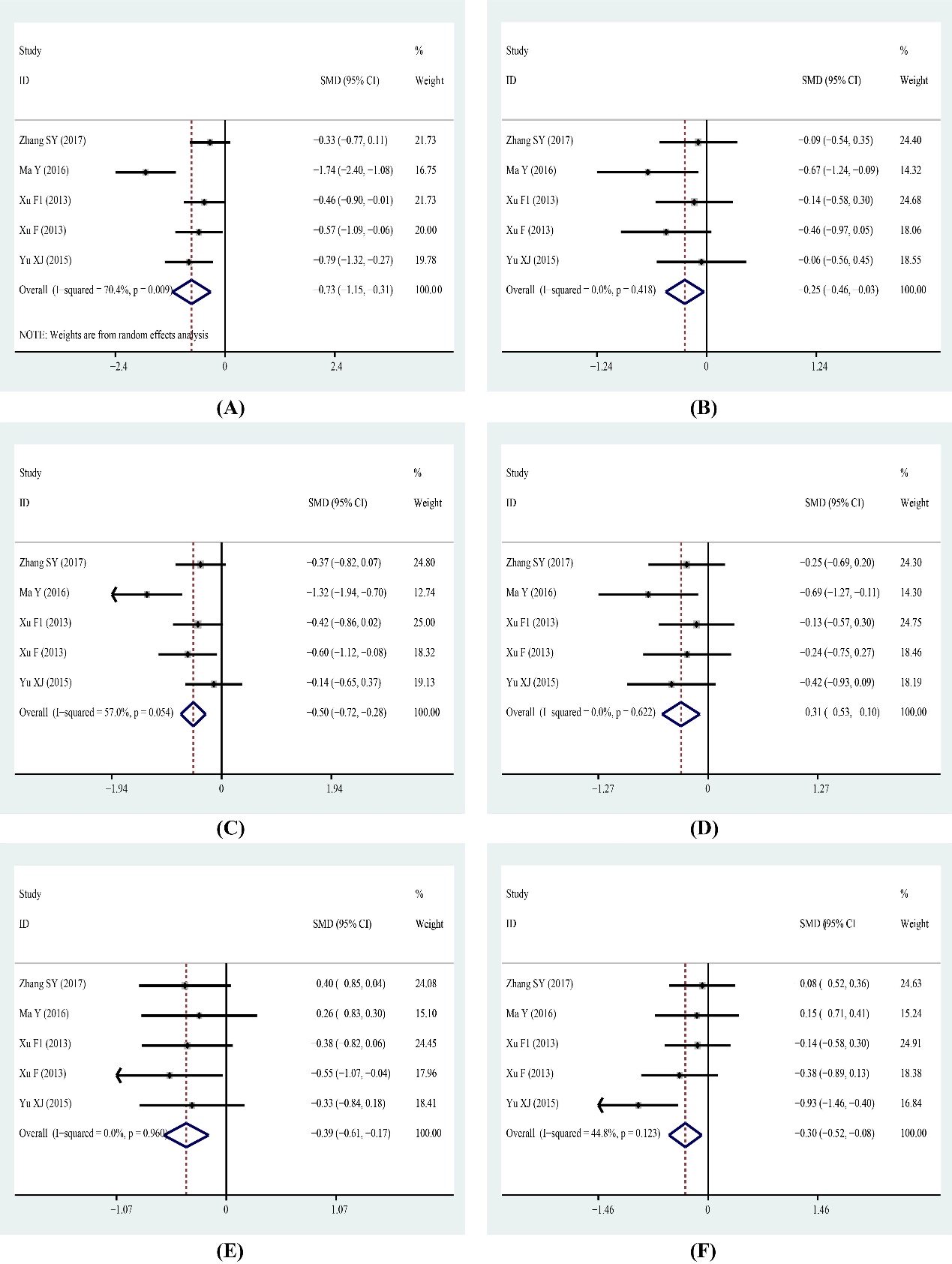


**Fig** **S8**. The forest plot of reduction of the SQ, FAT, ST, SE, SD, and DD scores (one-month follow-up) (ACE group versus ACU group). (A): reduction of the SQ score; (B): reduction of the FAT score; (C) reduction of the ST score; (D) reduction of the SE score; (D) reduction of the SD score; (F) reduction of the DD score.


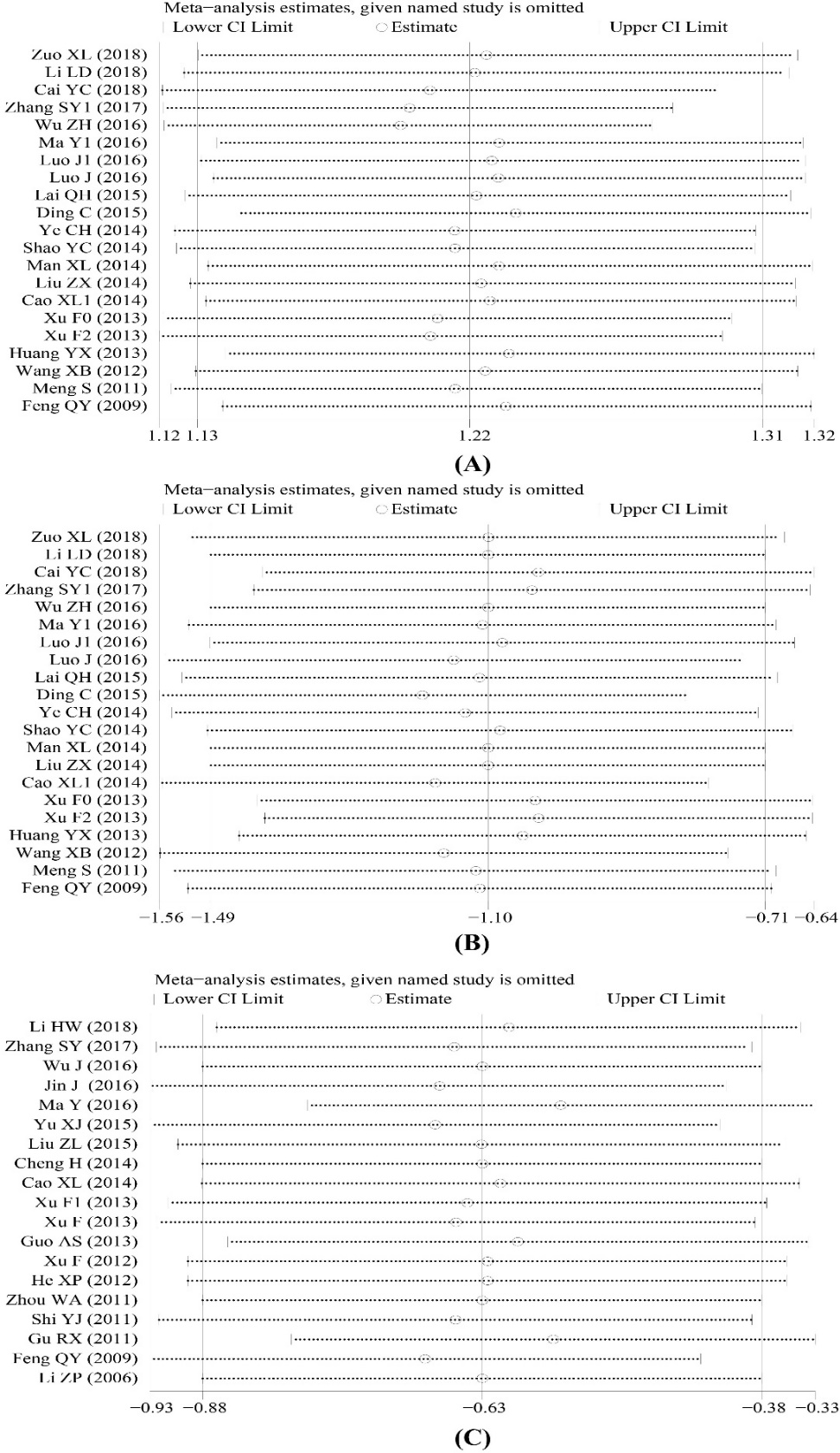


**Fig S9**. The forest plot of sensitivity analysis. (A): ACE group versus EZ group: clinical therapeutic effect; (B): ACE group versus EZ group: reduction of the PSQI score; (C) ACE group versus ACU group: reduction of the PSQI score.


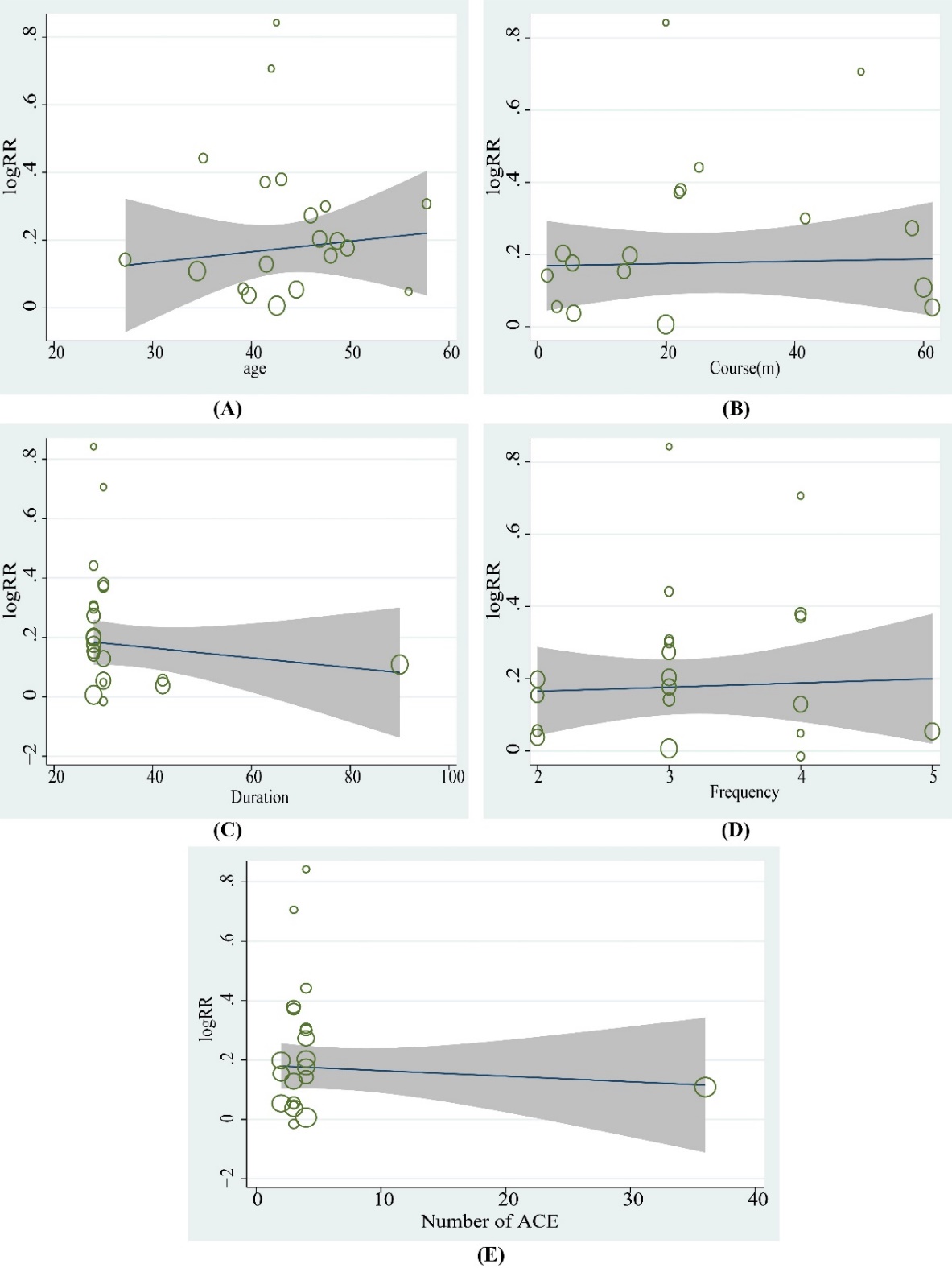


**Fig S10**. The graph of univariate meta regression analysis (ACE group versus EZ group: clinical therapeutic effect). The Y axis represents the quantitative synthesis results of clinical efficacy, and the X axis represents the influencing factors. Each circle represents a study, and the larger the circle, the higher the weight of the study. (A): mean age; (B): mean course of disease; (C): duration of treatment; (D): frequency of intervention; (E): number of intervention.


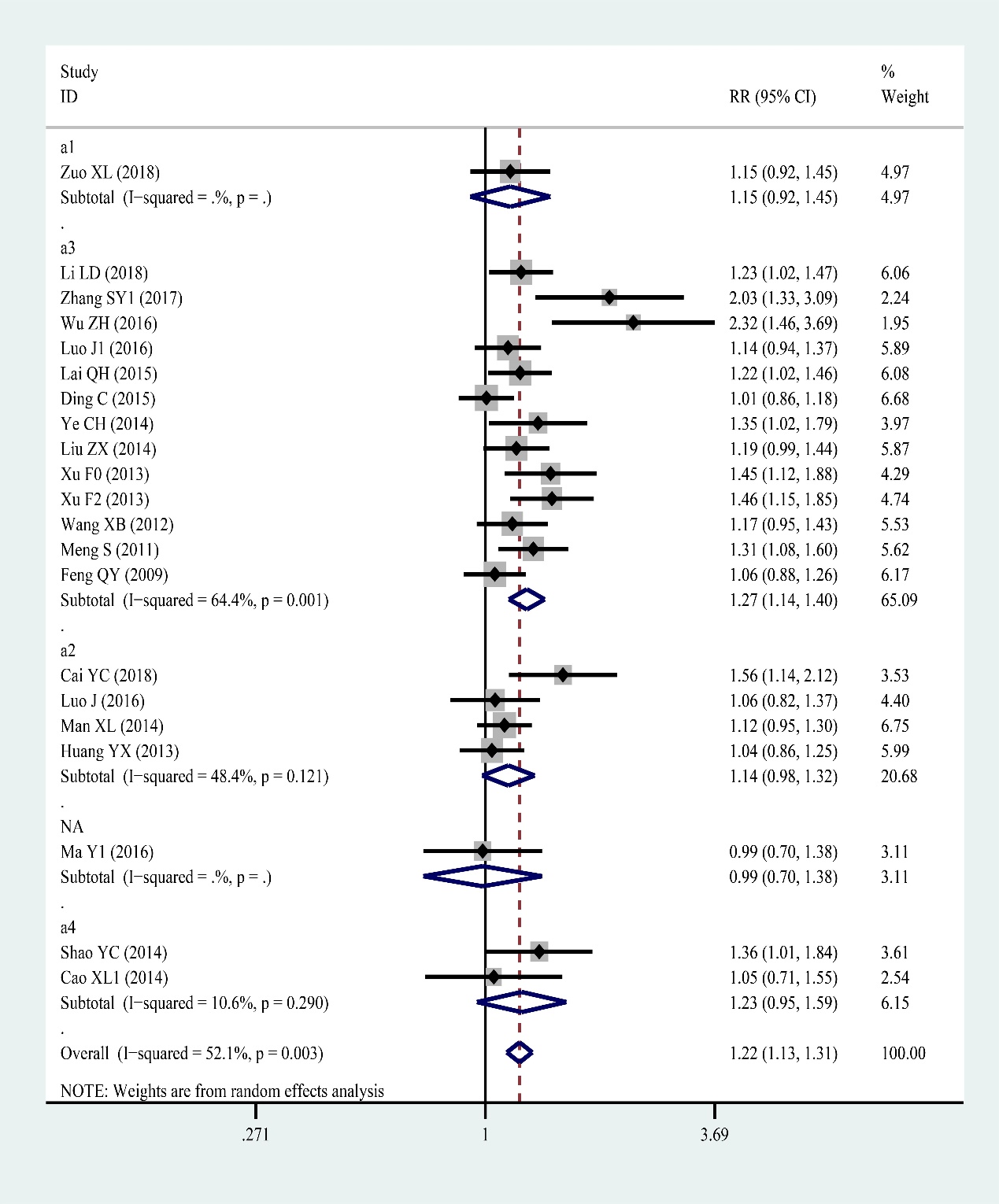


**Fig S11**. The forest plot of subgroup analysis by mean age (ACE group versus EZ group: clinical therapeutic effect). a1: 20≤mean age<30; a2: 30≤mean age<40; a3: 40≤mean age<50; a4: 50≤mean age<60; a5: mean age≥60; NA: unclear.


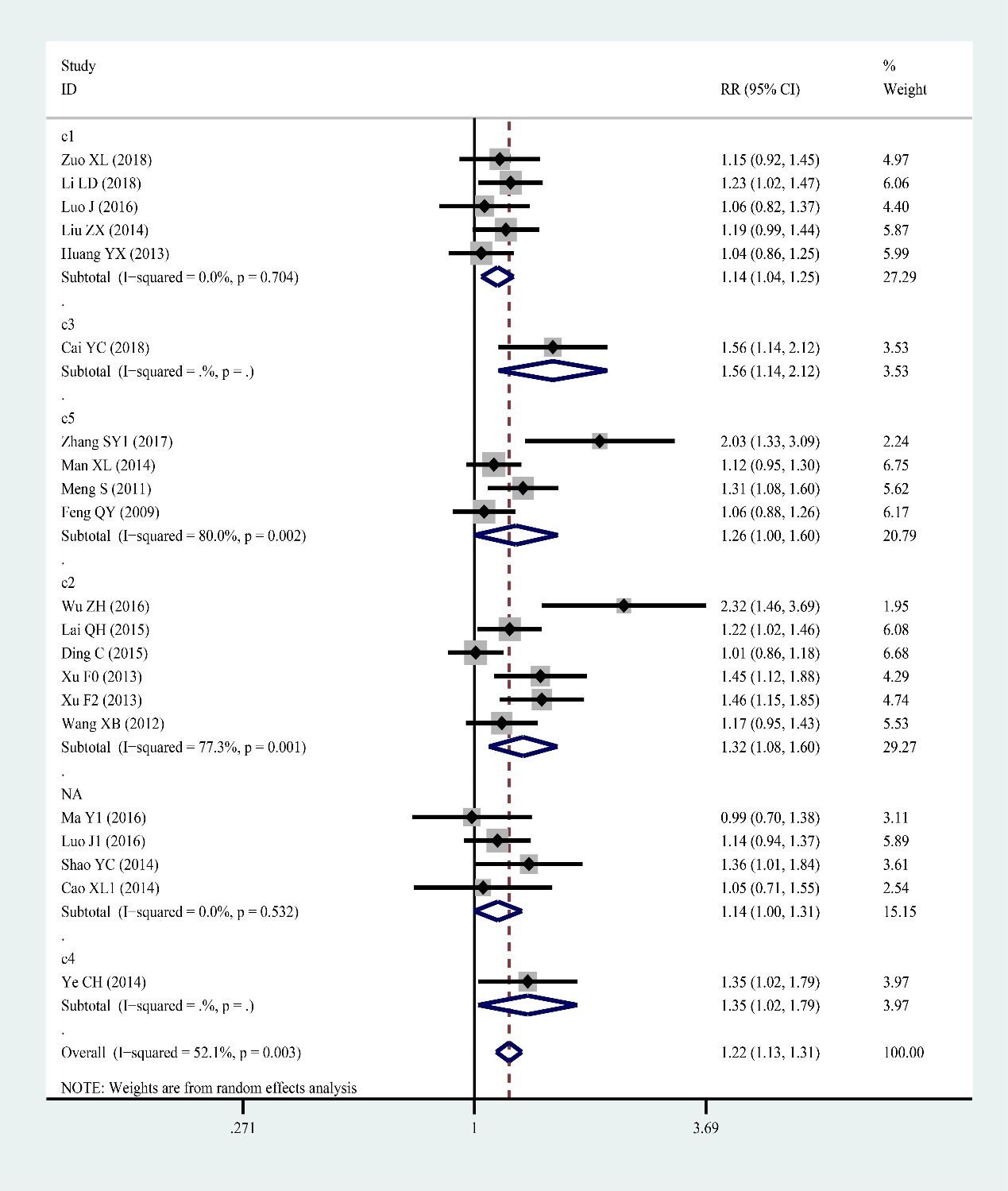


**Fig S12**. The forest plot of subgroup analysis by mean course of disease (ACE group versus EZ group: clinical therapeutic effect). c1: mean course <12 months; c2: 12 months ≤mean course<24 months; c3: 24 months ≤mean course <36 months; c4: 36 months ≤mean course <48 months; c5: mean course≥48 months; NA: unclear.


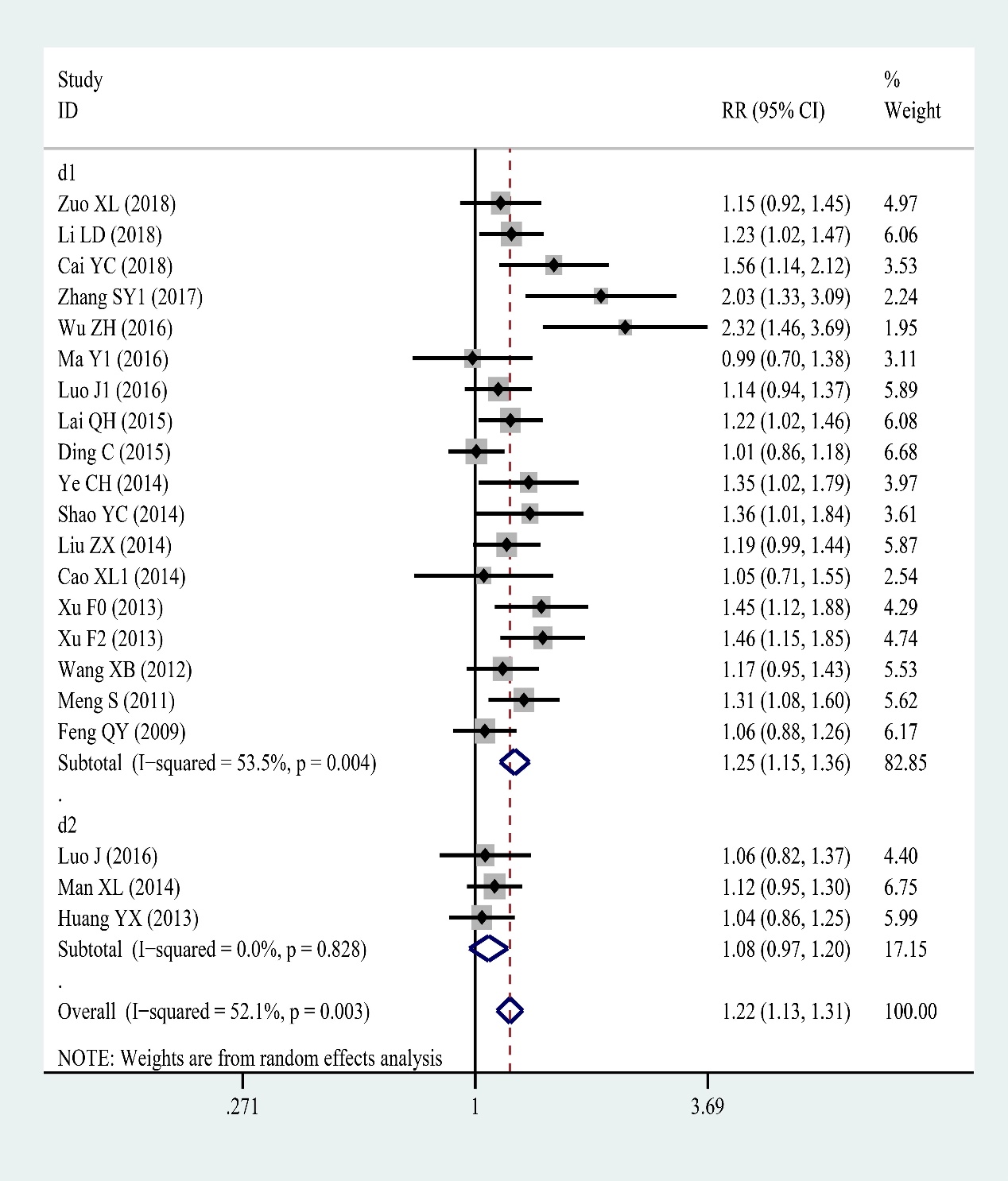


**Fig S13**. The forest plot of subgroup analysis by duration of treatment (ACE group versus EZ group: clinical therapeutic effect). d1: duration ≤30 days; d2: duration >30 days.


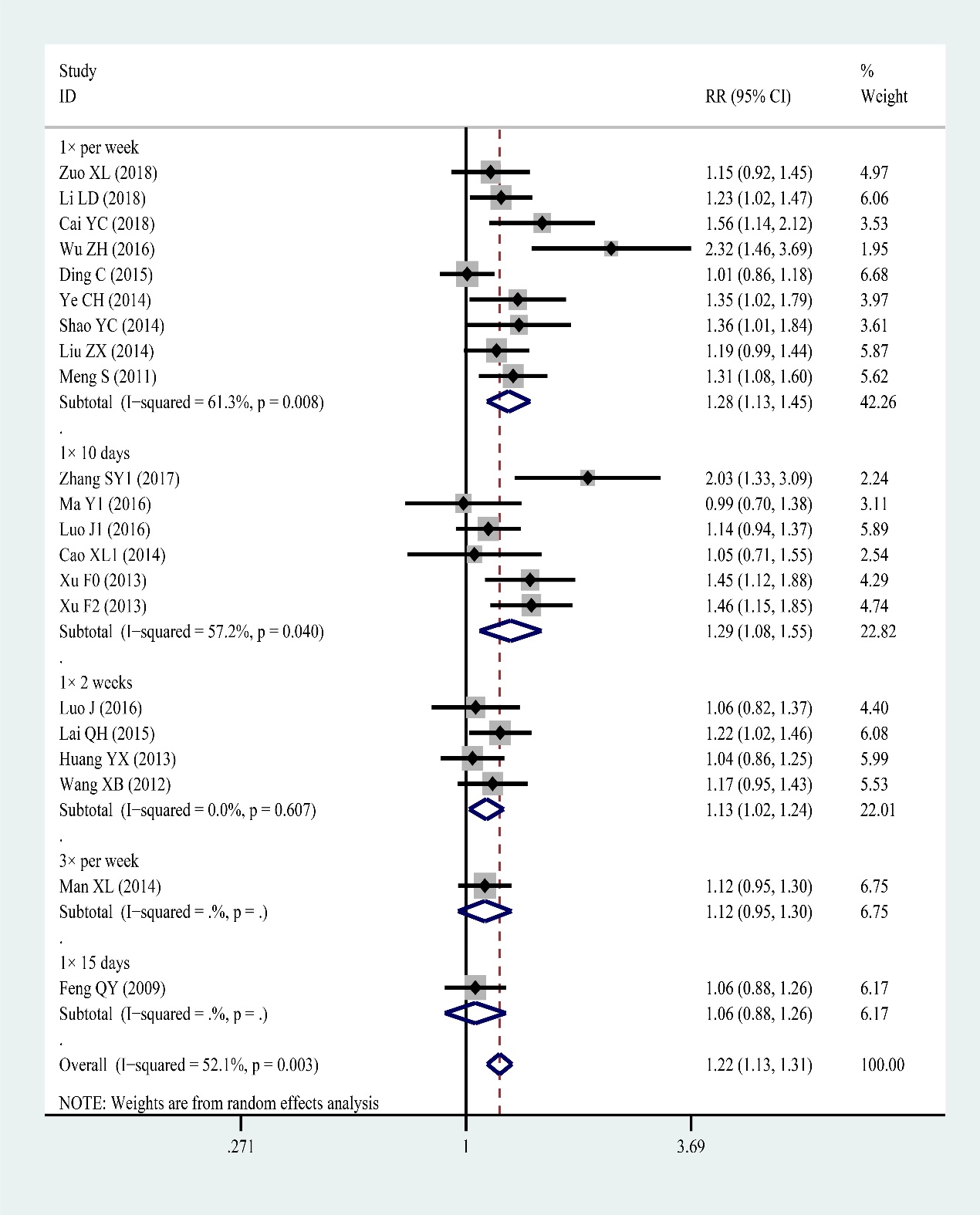


**Fig S14**. The forest plot of subgroup analysis by frequency of intervention (ACE group versus EZ group: clinical therapeutic effect).


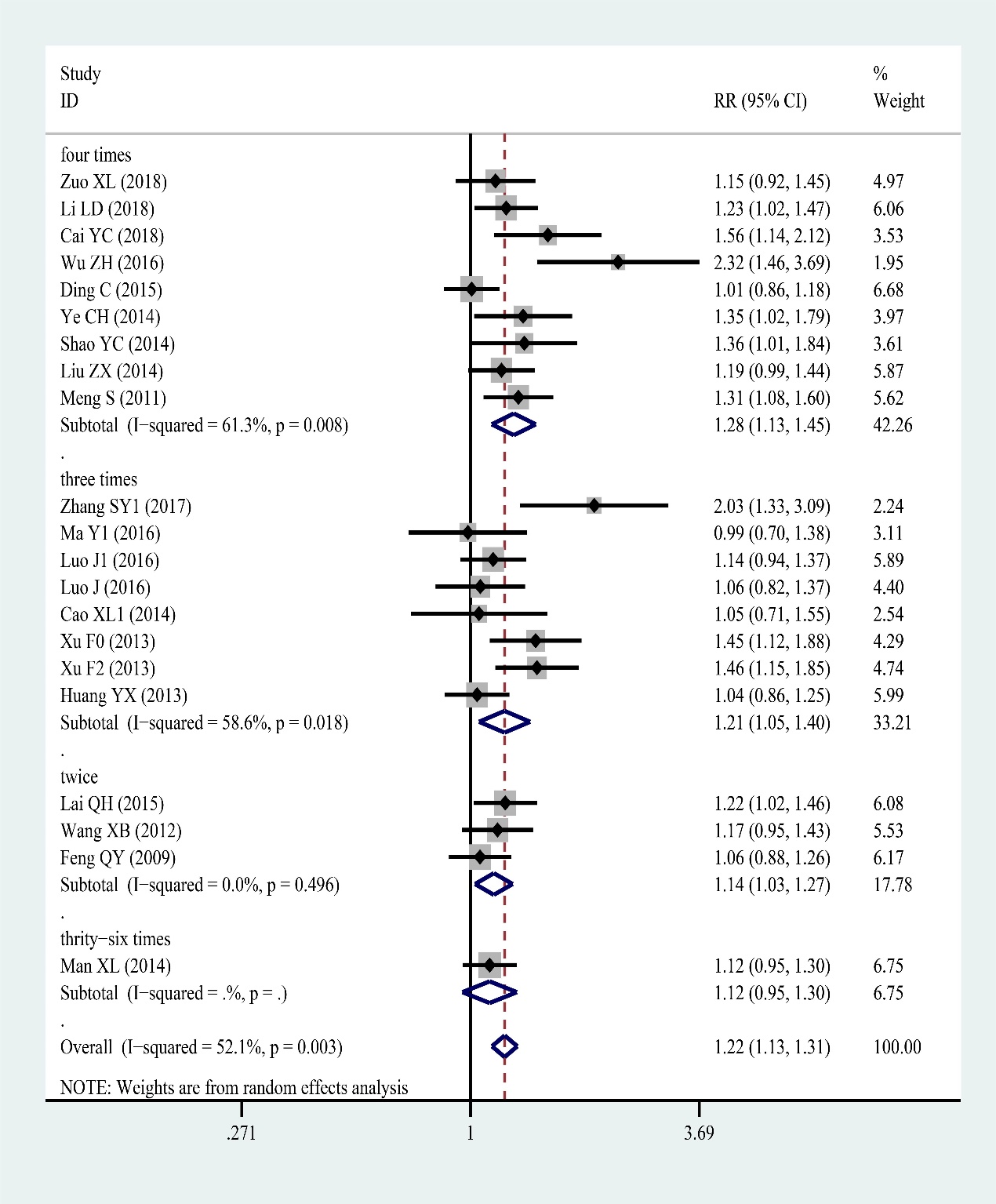


**Fig S15**. The forest plot of subgroup analysis by number of intervention (ACE group versus EZ group: clinical therapeutic effect).


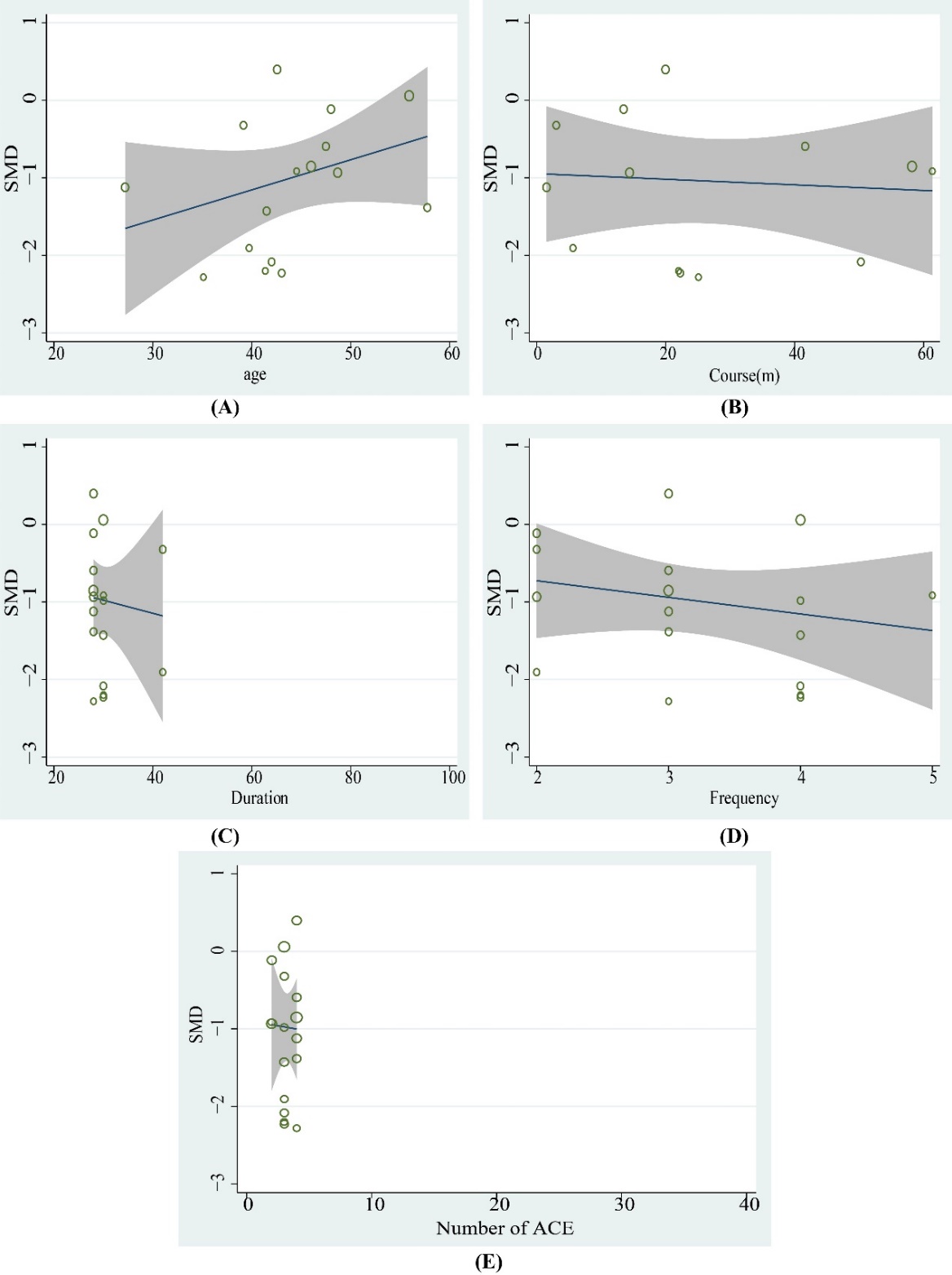


**Fig S16**. The graph of univariate meta regression analysis (ACE group versus EZ group: reduction of the PSQI score). The Y axis represents the quantitative synthesis results of reduction of the PSQI score, and the X axis represents the influencing factors. Each circle represents a study, and the larger the circle, the higher the weight of the study. (A): mean age; (B): mean course of disease; (C): duration of treatment; (D): frequency of intervention; (E): number of intervention.


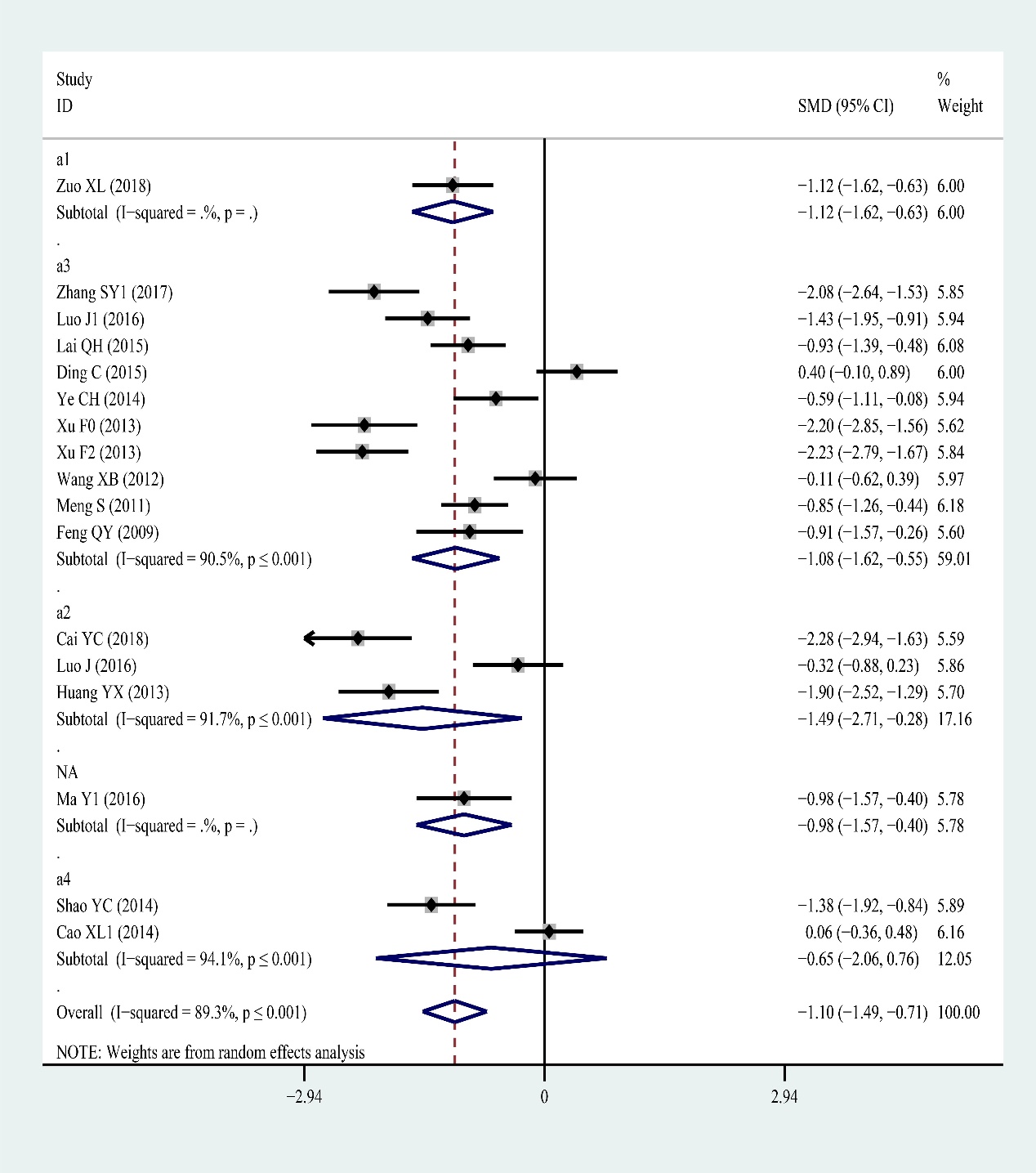


**Fig S17**. The forest plot of subgroup analysis by mean age (ACE group versus EZ group: reduction of the PSQI score). a1: 20≤mean age<30; a2: 30≤mean age<40; a3: 40≤mean age<50; a4: 50≤mean age<60; a5: mean age≥60; NA: unclear.


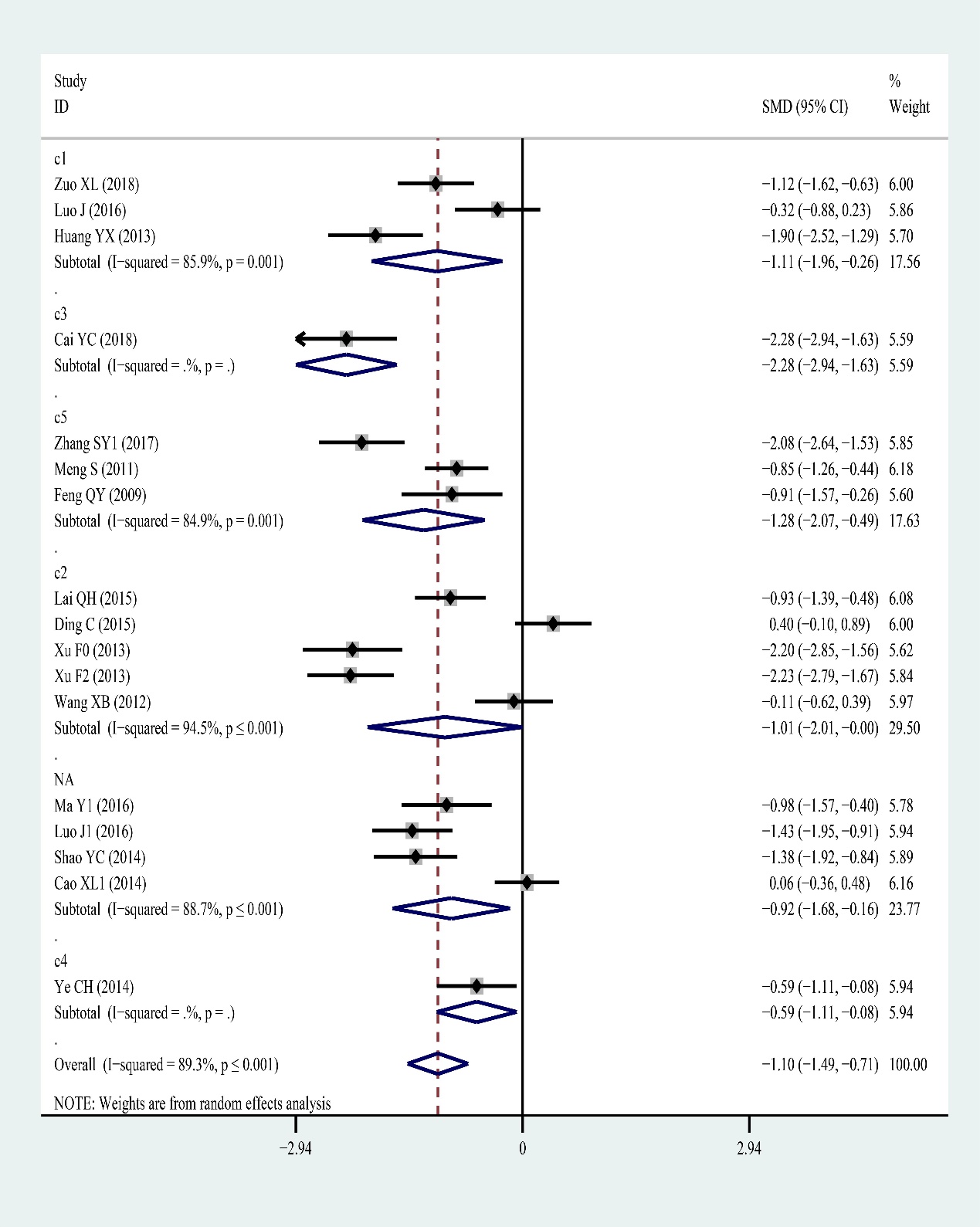


**Fig S18**. The forest plot of subgroup analysis by mean course of disease (ACE group versus EZ group: reduction of the PSQI score). c1: mean course <12 months; c2: 12 months ≤mean course<24 months; c3: 24 months ≤mean course <36 months; c4: 36 months ≤mean course <48 months; c5: mean course≥48 months; NA: unclear.


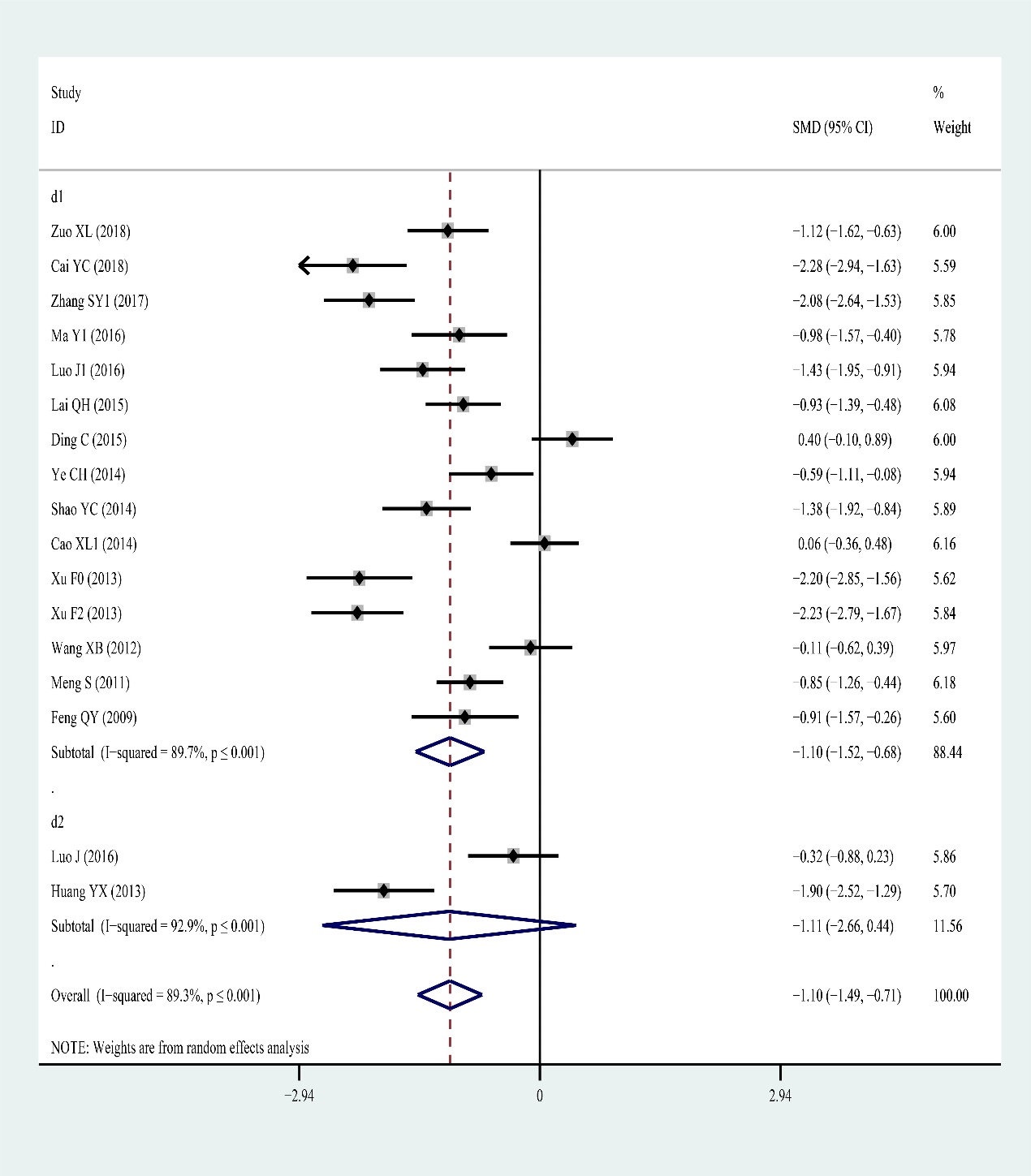


**Fig S19**. The forest plot of subgroup analysis by duration of treatment (ACE group versus EZ group: reduction of the PSQI score). d1: duration ≤30 days; d2: duration >30 days.


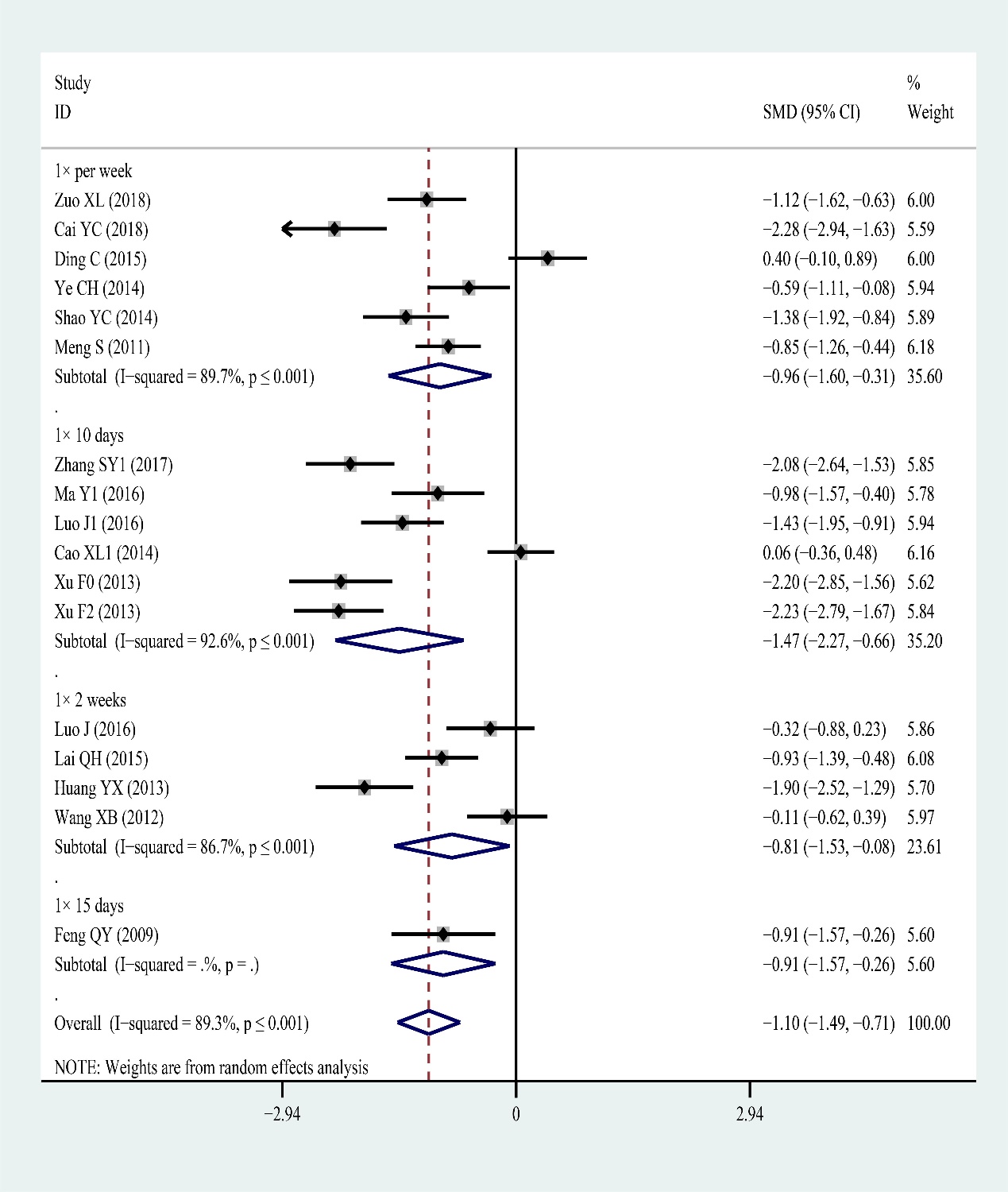


**Fig S20**. The forest plot of subgroup analysis by frequency of intervention (ACE group versus EZ group: reduction of the PSQI score).


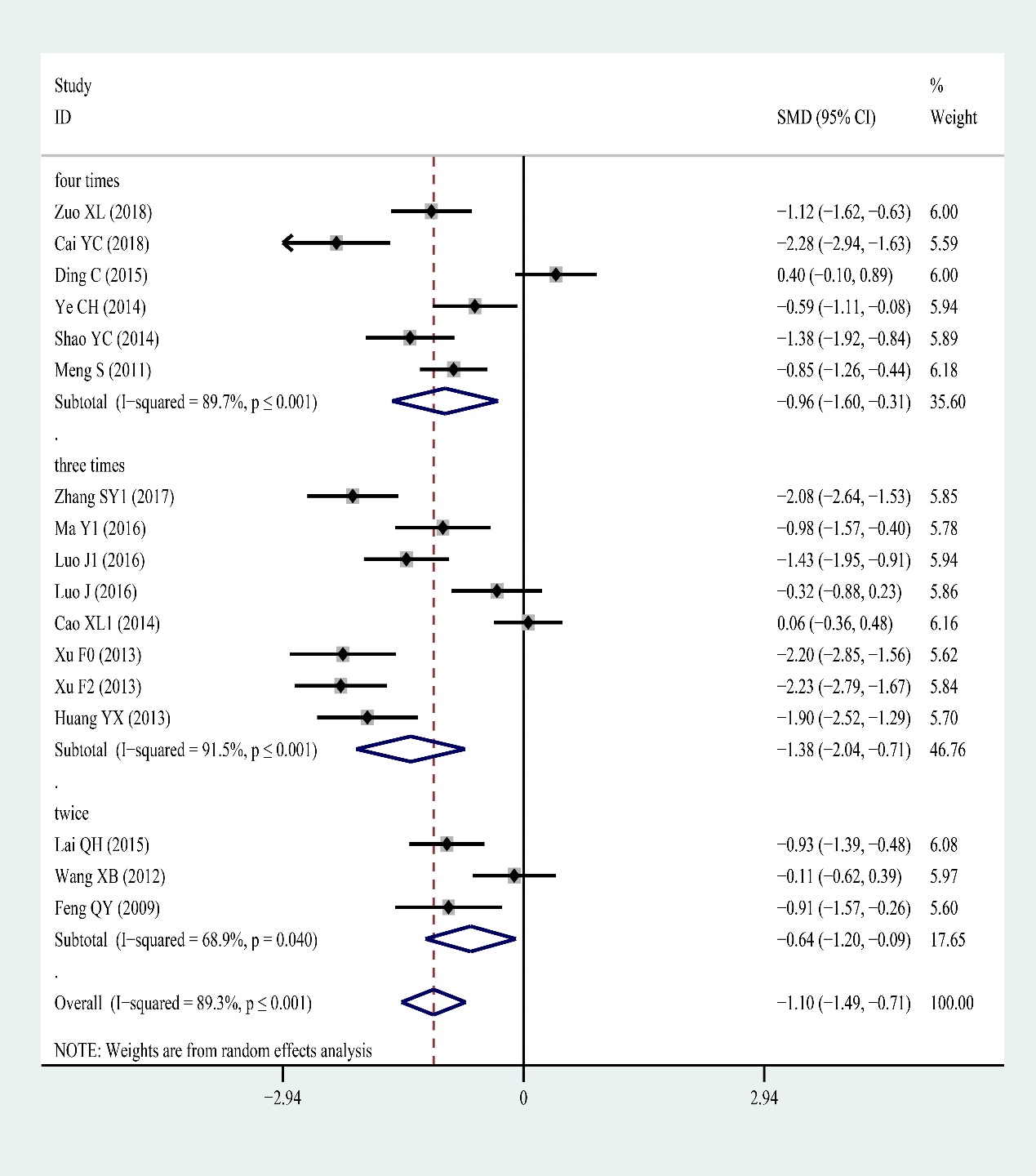


**Fig** **S21**. The forest plot of subgroup analysis by number of intervention (ACE group versus EZ group: reduction of the PSQI score).


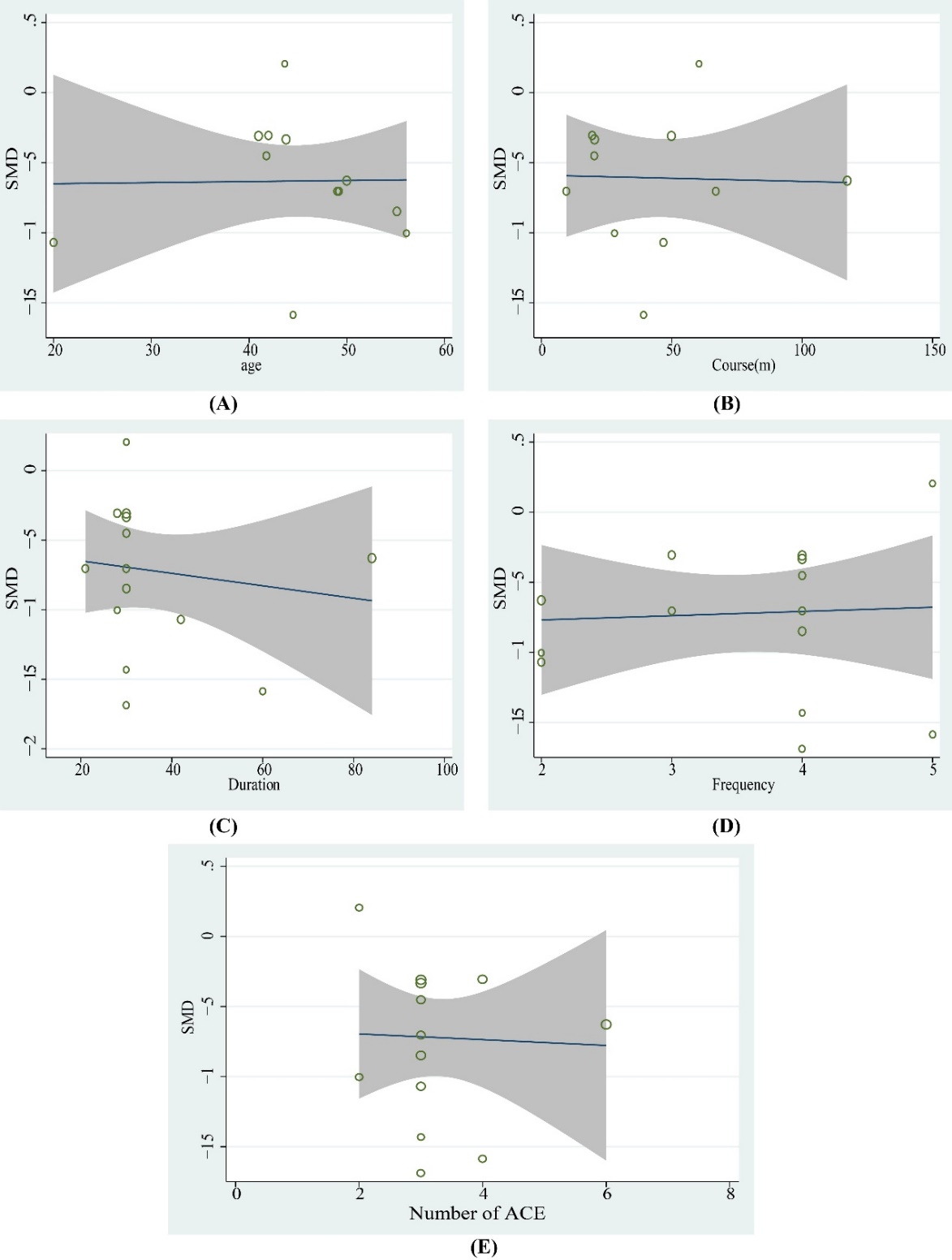


**Fig** **S22**. The graph of univariate meta regression analysis (ACE group versus ACU group: reduction of the PSQI score). The Y axis represents the quantitative synthesis results of reduction of PSQI score, and the X axis represents the influencing factors. Each circle represents a study, and the larger the circle, the higher the weight of the study. (A): mean age; (B): mean course of disease; (C): duration of treatment; (D): frequency of intervention; (E): number of intervention.


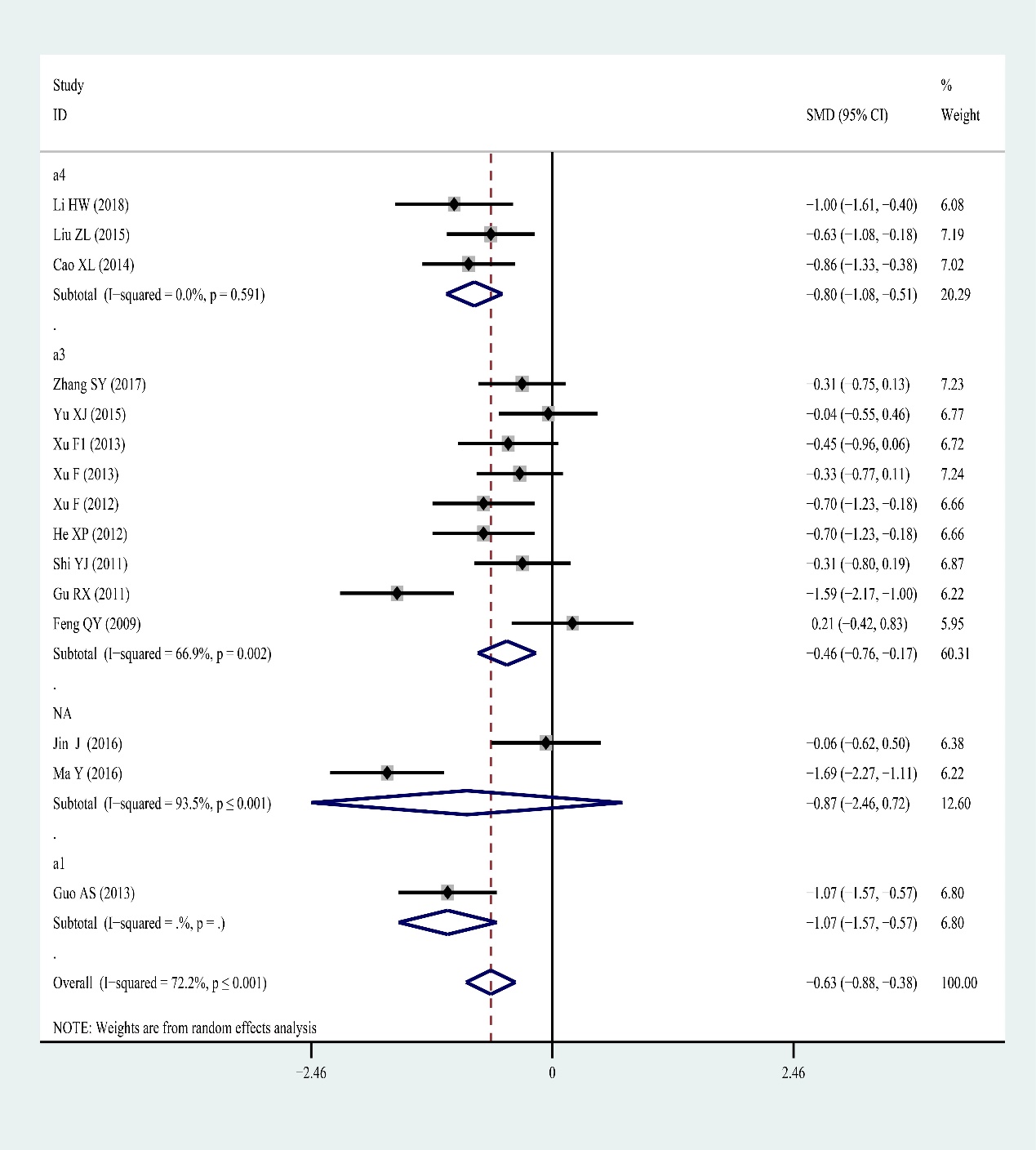


**Fig** **S23**. The forest plot of subgroup analysis by mean age (ACE group versus ACU group: reduction of the PSQI score). a1: 20≤mean age<30; a2: 30≤mean age<40; a3: 40≤mean age<50; a4: 50≤mean age<60; a5: mean age≥60; NA: unclear.


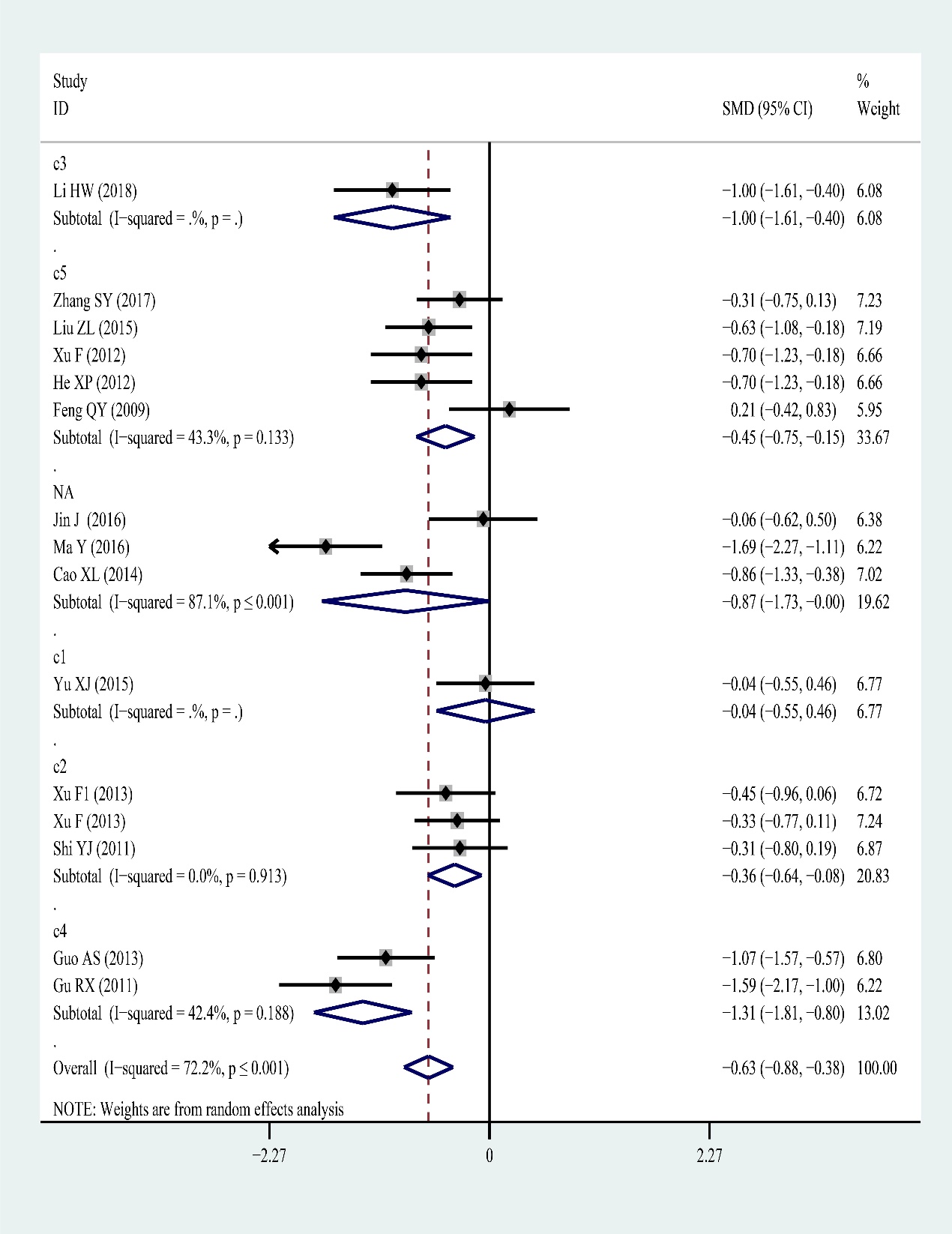


**Fig** **S24**. The forest plot of subgroup analysis by mean course of disease (ACE group versus ACU group: reduction of the PSQI score). c1: mean course <12 months; c2: 12 months ≤mean course<24 months; c3: 24 months ≤mean course <36 months; c4: 36 months ≤mean course <48 months; c5: mean course≥48 months; NA: unclear.


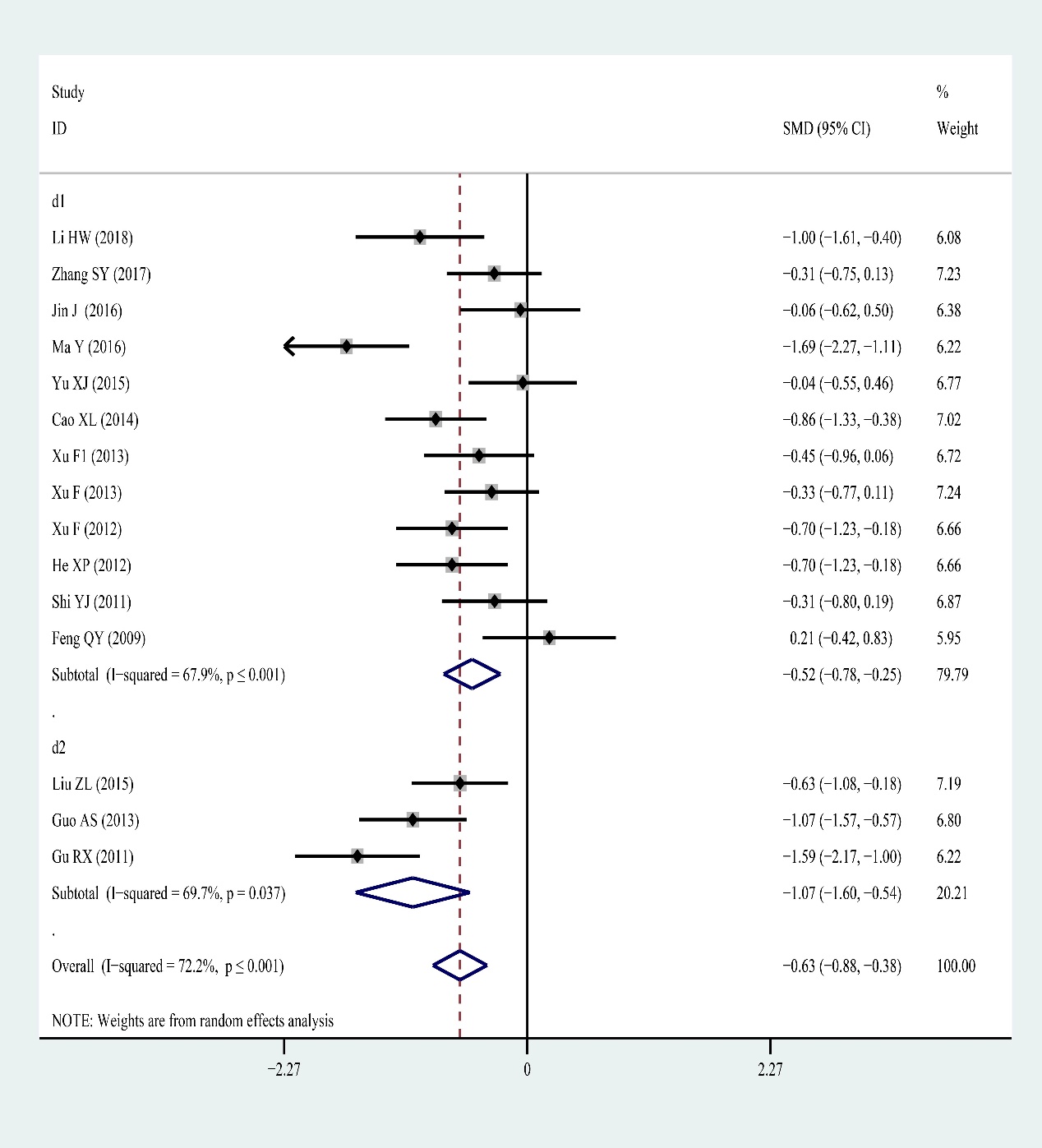


**Fig** **S25**. The forest plot of subgroup analysis by duration of treatment (ACE group versus ACU group: reduction of the PSQI score). d1: duration ≤30 days; d2: duration >30 days.


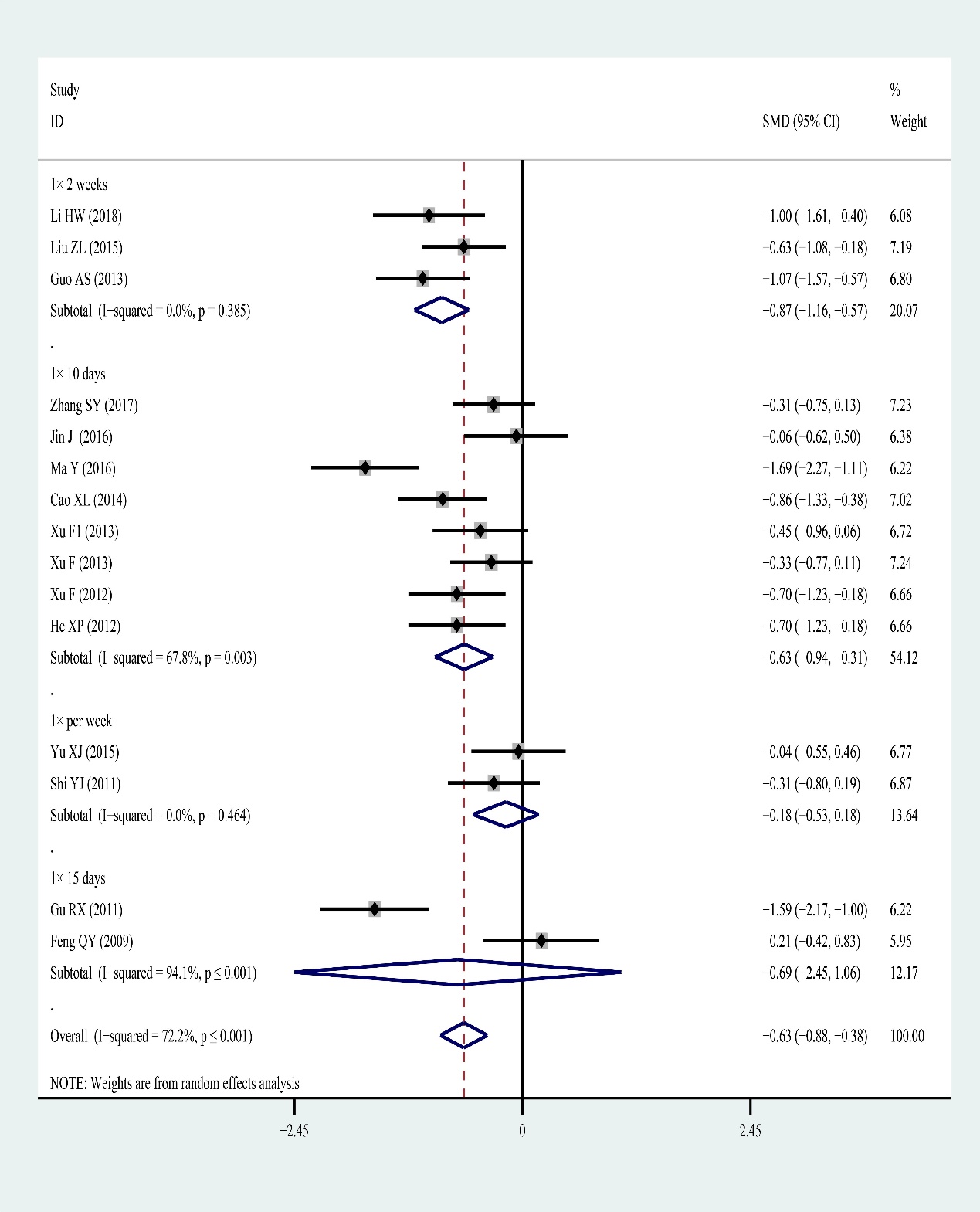


**Fig** **S26**. The forest plot of subgroup analysis by frequency of intervention (ACE group versus ACU group: reduction of the PSQI score).


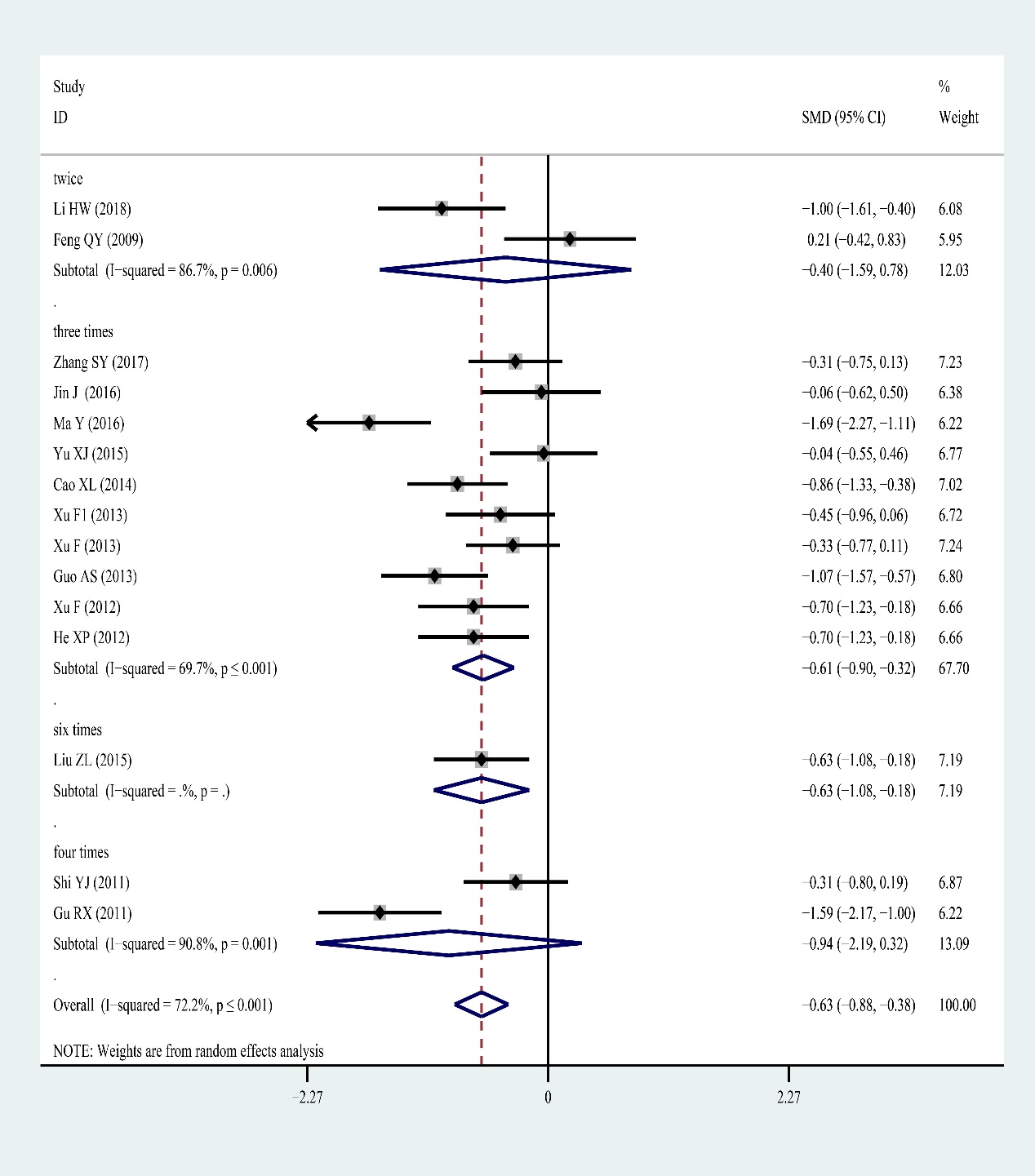


**Fig** **S27**. The forest plot of subgroup analysis by number of intervention (ACE group versus ACU group: reduction of the PSQI score).
